# Host Translational Control by Stress Granules Promotes *Mycobacterium tuberculosis* Pathogenesis

**DOI:** 10.1002/mco2.70479

**Published:** 2025-11-10

**Authors:** Jaewhan Kim, Sang‐Hun Son, Ji‐Ae Choi, Junghwan Lee, Seoyeon Jo, Soo‐Na Cho, Doan Tam Nguyen, Doyi Son, Kee K. Kim, Chang‐Hwa Song

**Affiliations:** ^1^ Department of Medical Science, College of Medicine Chungnam National University Daejeon Republic of Korea; ^2^ Department of Biochemistry, College of Natural Sciences Chungnam National University Daejeon Republic of Korea; ^3^ Department of Microbiology, College of Medicine Chungnam National University Daejeon Republic of Korea

**Keywords:** ATP, mitochondria, *Mycobacterium tuberculosis*, stress granule, tuberculosis

## Abstract

Stress granules (SGs) are cytoplasmic condensates that regulate mRNA translation and signaling in response to stress. Although SGs have been widely studied in antiviral responses, their function in bacterial infections is not well understood. Here, we demonstrate that SGs promote *Mycobacterium tuberculosis* (Mtb) pathogenesis by suppressing mitochondrial metabolism and innate immunity. Quantitative proteomics revealed that Mtb‐induced SGs sequester mTORC1 and suppress cap‐dependent mRNA translation. This leads to decreased expression of proteins necessary for mitochondrial respiration and immune activation in bone marrow‐derived macrophages (BMDMs). Disrupting SG assembly restored mTORC1 signaling, enhanced oxidative phosphorylation, and increased the production of antimicrobial mediators, such as reactive oxygen species, nitric oxide, and proinflammatory cytokines. This restricted intracellular Mtb growth in vitro and in vivo. Mechanistically, intracellular ATP depletion triggered by Mtb phagocytosis was sufficient to drive SG formation, linking energy stress to translational repression. Furthermore, SGs captured Ndufa12, a complex I subunit, thereby impairing mitochondrial electron transport and ATP production. These findings identify SGs as key regulators that couple translational arrest to metabolic and immune suppression, enabling Mtb persistence. Targeting SG formation or function could be a host‐directed strategy to restore mitochondrial activity and strengthen immune responses against Mtb infection.

## Graphical abstract

During *Mycobacterium tuberculosis* infection, stress granules (SGs) formed in macrophages sequester key signaling and metabolic regulators such as mTORC1 components (mTOR, Raptor) and mitochondrial complex I subunits (Ndufa12), leading to suppressed cap‐dependent translation, reduced mitochondrial activity, and impaired innate immune responses. In contrast, SG‐deficient macrophages (G3bp1/2 dKD) maintain mTORC1 activity and mitochondrial function, resulting in enhanced immune activation and restricted Mtb survival both in vitro and in vivo. These findings reveal SGs as critical regulators of host cell metabolism and immunity during Mtb infection.

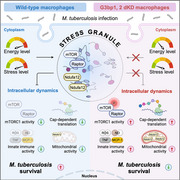

## 1 Introduction

Tuberculosis (TB) is a major public health concern, with 5%–15% of *Mycobacterium tuberculosis* (Mtb) infections progressing to active disease, whereas the rest have the potential to develop active TB throughout their lifetime [1]. Mtb primarily targets macrophages [2] and deploys various strategies, including inhibition of phagolysosomal fusion and apoptosis, detoxification of reactive oxygen species (ROS), and induction of necrosis, to facilitate bacterial dissemination to evade immune responses [3]. However, the precise mechanisms underlying Mtb immune evasion remain unclear, emphasizing the need to understand the factors contributing to Mtb intracellular survival.

During infection, host cells are exposed to various cellular stresses that trigger the formation of stress granules (SGs) [4, 5]. SGs are dynamic ribonucleoprotein condensates containing untranslated mRNAs, translation initiation factors, and proteins [6, 7]. They play crucial roles in mRNA metabolism and fine‐tune the physiological responses of immune cells [8]. Furthermore, SGs are considered a survival mechanism employed by cells, as they reduce unnecessary mRNA translation to conserve energy for cellular stress–relief mechanisms [9]. While SGs are known to play dual roles during viral infections—either enhancing antiviral defenses or suppressing host immunity to favor viral replication—their functions during bacterial infection are less clear [10, 11, 12]. The pathogen‐specific regulation suggests that SGs might act as critical modulators of infection outcomes [13]. The roles of SGs during Mtb infection have not been fully characterized, raising questions about how SGs influence innate immune pathways and bacterial survival within macrophages.

Certain Mtb strains, such as H37Rv and Erdman, that induce lysosomal damage have been shown to induce the formation of SGs, thereby inhibiting Mtb survival in macrophages [14, 15]. In our previous studies, we have shown that Mtb infection activates the integrated stress response (ISR), namely, the phosphorylation of Eif2α, a key initiating factor of SG assembly, regardless of lysosomal damage [16, 17]. SGs have also been linked to the enhancement of type I interferon (IFN) responses [18], and plasmacytoid dendritic cell (pDC)‐derived type I IFN increases susceptibility to Mtb by impairing macrophage‐mediated Mtb control [19]. These findings emphasize the need for a detailed mechanistic understanding of how SGs regulate immune responses during Mtb infection.

Several studies have shown that mammalian target of rapamycin (mTOR), a critical regulatory protein involved in various intracellular signaling pathways and immune cell activation [20, 21, 22], is also regulated by SGs [23, 24]. Mitochondrial energy metabolism controlled by mTOR is crucial for macrophage survival and antimicrobial activity [25]. Notably, intracellular ATP levels have recently been shown to be critical determinants of SG formation [26]. These studies collectively suggest that SG‐mediated regulation of cellular metabolism and immune responses could play a pivotal role in determining TB pathogenesis.

Here, we demonstrate that Mtb infection triggers energy depletion and SG formation, which suppress global mRNA translation, mitochondrial respiration, and innate immune responses in macrophages. Disruption of SGs restores these activities and markedly restricts Mtb survival in infected cells and mouse models. These findings highlight SG dynamics as key modulators of host energy metabolism and immune defense during Mtb infection, suggesting that targeting SGs could provide a novel strategy for host‐directed TB therapy.

## 2 Results

### 2.1 Decreased Expression of Immune System‐Associated Proteins in Mtb‐Infected Macrophages

Microbial infection alters host gene and protein expression [27, 28]. To identify proteins critical for anti‐Mtb responses, we performed proteomic analysis of infected bone marrow‐derived macrophages (BMDMs; Figure S1A). A total of 756 proteins were significantly changed, giving infected cells distinct expression profiles compared with uninfected controls (UNs; Figure S1B and Table S1). Over 300 proteins were upregulated (Figure S1C,D), and immune‐related proteins increased at 12 and 24 h postinfection (hpi; Figure S1F,G). However, compared with UN, proteins involved in immune processes decreased at 24 hpi but not at 12 hpi (Figure S1H,I). These results suggest that Mtb infection initially activates immune responses, which decline with prolonged infection.

### 2.2 Mtb Infection Induces SG Formation Both In Vitro and In Vivo

Between 12 and 24 hpi, 124 proteins increased while 97 decreased, indicating time‐dependent proteomic changes in infected macrophages (Figure S1E). Functional annotation of 32 proteins with time‐dependent upregulation revealed a significant enrichment in RNA‐binding functions (Figure S2A,B), essential for stress responses and innate immunity [29]. Given the evidences that Mtb infection induces stress in macrophages and that RNA‐binding proteins (RBPs) are associated with SGs [30, 31], we analyzed SG‐associated proteins and found their expression markedly increased at 12 and 24 hpi (Figure S2C).

**FIGURE 1:**
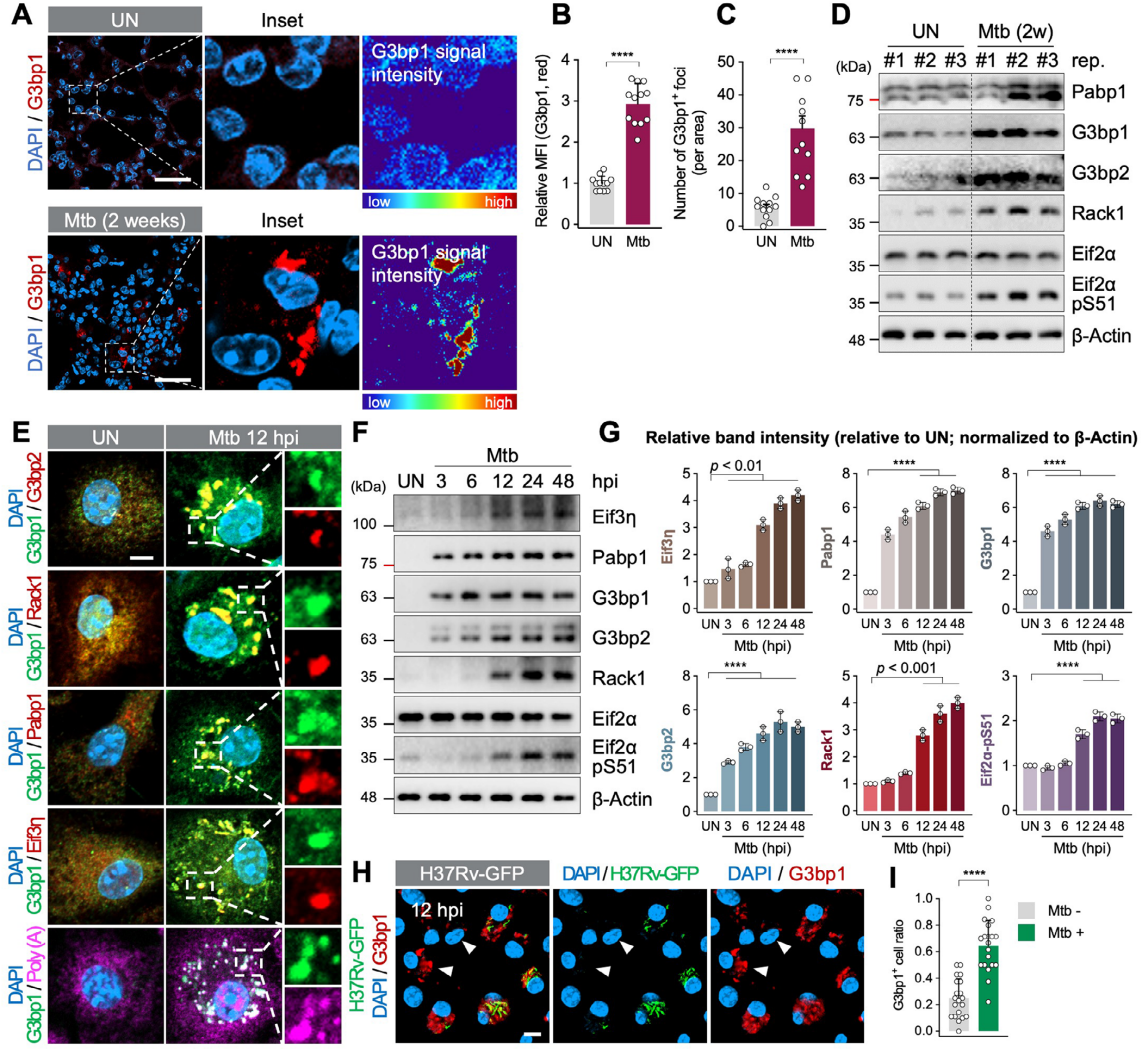
Mtb infection triggers robust SG formation in macrophages. (A) Immunofluorescence of lung tissues from UN and infected mice. G3bp1 fluorescence intensity was visualized in rainbow color to highlight differences in signal intensity. Scale bar indicates 50 µm. (B, C) Quantification of G3bp1 mean fluorescence intensity (MFI) (B) and G3bp1‐positive foci per area (C) from (A). Data are presented as mean ± SEM. ****p < 0.0001. *N* = 12. (D) Immunoblot of SG‐related proteins in lung lysates of UN and Mtb‐infected mice. (E) Immunofluorescence of SG markers in BMDMs infected with Mtb (MOI 1, 12 hpi). Scale bar indicates 10 µm. (F) Immunoblot of SG proteins in BMDMs at indicated times (MOI 1). (G) Quantification of relative band intensities from (F), normalized to β‐Actin. Data are presented as relative band intensities compared to UN. ****p < 0.0001. *N* = 3. (H) Immunofluorescence G3bp1 in H37Rv‐GFP infected BMDMs (MOI 1, 12 hpi). White arrowhead indicates H37Rv‐GFP‐negative cells. Scale bar indicates 10 µm. (I) Quantification of G3bp1‐positive cell ratio in H37Rv‐GFP^−^ cells or H37Rv‐GFP^+^ cells from (H). ****p < 0.0001. *N* = 20.

To test SG formation in vivo, mice were infected with Mtb for 2 weeks. Lung tissues showed robust SG formation, confirmed by established SG markers [32] (Figure 1A–D). In BMDMs, SG markers also increased in a time‐dependent manner and their expression was saturated at 24 hpi (Figure 1E–G). Notably, even uninfected bystander cells also developed modest SGs, while cells that phagocytosed Mtb showed stronger responses (Figure 1H,I). These results indicate that intracellular Mtb infection strongly triggers SG assembly.

### 2.3 SGs Inhibit mRNA Translation Upon Mtb Infection

**FIGURE 2:**
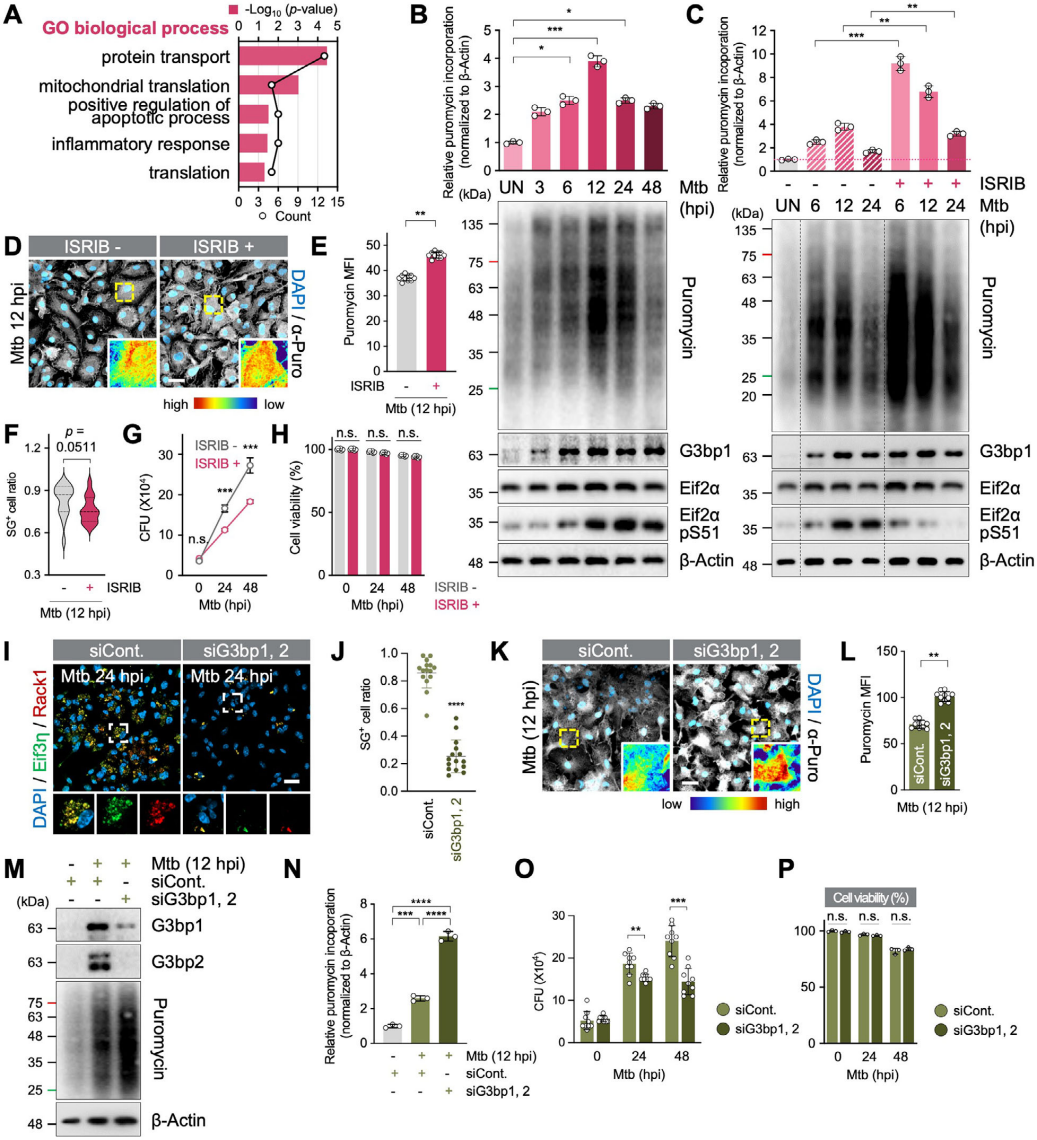
SGs suppress host protein synthesis to support Mtb survival. (A) GO analysis of 97 proteins with log_2_ ratio (24 hpi/12 hpi) < −1.0 from (Figure S1E); top five GO categories are shown, ranked by *p* value. (B) Ribopuromycylation assay of de novo protein synthesis in Mtb‐infected BMDMs (MOI 1). *p < 0.05, ***p < 0.001. *N* = 3. (C) Ribopuromycylation assay in ISRIB‐treated or untreated BMDMs, during Mtb infection (MOI 1). **p < 0.01, ***p < 0.001. *N* = 3. (D) Puromycin immunofluorescence in infected BMDMs ± ISRIB (MOI 1, 12 hpi). Puromycin signal intensity was visualized using a rainbow color scale. Scale bar indicates 20 µm. (E) MFI of the puromycin from (D). Data are presented as mean ± SEM. **p < 0.01. *N* = 10. (F) SG‐positive BMDM ratio with or without ISRIB (MOI 1, 12 hpi) were plotted as violin plot with median and quartiles. (G) Intracellular survival of Mtb in ISRIB‐treated or untreated BMDMs (MOI 1). Data are presented as mean ± SEM. n.s., nonsignificant, ***p < 0.001, compared within the same time point. *N* = 9. (H) Cell viability (LDH assay) of ISRIB‐treated or untreated BMDMs (MOI 1). Relative to 0 hpi ISRIB ‐. n.s., nonsignificant; compared within the same time point. *N* = 5. (I) Immunofluorescence of SGs in WT (siCont.) and G3bp1/2‐dKD (siG3bp1,2) BMDMs (MOI 1, 24 hpi). Scale bar indicates 20 µm. (J) Quantification of SG‐positive cells from (I). Both Eif3η‐ and Rack1‐positive puncta are considered as SGs. ****p < 0.0001, compared to siCont. *N* = 15. (K) Immunofluorescence of puromycin in WT and SG^neg^ BMDMs (MOI 1, 12 hpi). Rainbow color gradient indicates puromycin signal intensity. Scale bar indicates 20 µm. (L) MFI of puromycin from (K). **p < 0.01. *N* = 10. (M) Ribopuromycylation assay in Mtb‐infected SG^neg^ BMDMs (MOI 1, 12 hpi). (N) Relative puromycin intensity from (M), normalized to β‐Actin. ***p < 0.001, ****p < 0.0001. *N* = 3. (O) Intracellular survival of Mtb in WT and SG^neg^ BMDMs (MOI 1). Data are presented as mean ± SEM. **p < 0.01, ***p < 0.001. *N* = 9. (P) Cell viability (LDH assay) of WT and SG^neg^ BMDMs (MOI 1). Relative to 0 hpi siControl (siCont.). n.s., nonsignificant; compared within the same time point. *N* = 3.

Because SG formation peaked at 24 hpi (Figure 1G; see also Figure 6B), we hypothesized that SG‐associated or SG‐affected proteins might differ between 12 and 24 hpi. Comparative Gene Ontology (GO) analysis of 97 proteins reduced at 24 hpi, compared to 12 hpi, revealed enrichment in protein transport, mitochondrial translation, apoptosis, and inflammatory responses (Figure 2A; see also Figure S1E). The reduced abundance of proteins associated with apoptosis and inflammation suggests a weakened innate immune response, supporting TB pathogenesis [33].

Translation‐related proteins decreased from 12 to 24 hpi, implying that saturated SGs might suppress mRNA translation. To test this hypothesis, we performed a ribopuromycylation assay to profile mRNA translation patterns during Mtb infection. We found that translation increased up to 12 hpi, likely reflecting an early immune response, then declined sharply, while G3bp1 and Eif2α‐pS51 levels remained increased (Figure 2B). Inhibition of the ISR with ISR inhibitor (ISRIB) restored translation and lowered the proportion of SG‐positive cells (Figure 2C–F). The ISRIB‐induced upregulation of mRNA translation led to a reduction in Mtb growth in macrophages without affecting host cell death (Figure 2G,H). These data suggest that SGs promote Mtb survival by dampening host mRNA translation.

Next, to examine whether SGs formed during Mtb infection inhibit mRNA translation, we used G3bp1 and G3bp2 double knockdown (dKD; SG^neg^) macrophages, in which SG formation was strongly inhibited (Figure 2I,J). Mtb‐infected SG^neg^ macrophages showed a marked increase in mRNA translation (Figure 2K–N) and suppressed Mtb survival without affecting host cell viability (Figure 2O,P). Enhanced translation was not observed in uninfected SG^neg^ macrophages, indicating that the effect is specific to the disruption of infection‐induced SGs rather than a nonspecific consequence of G3bp1 and G3bp2 depletion (Figure S5B–D). Together, these findings demonstrate that Mtb‐induced SGs inhibit mRNA translation, supporting Mtb survival in macrophages.

### 2.4 SGs Inhibit Cap‐Dependent Translation by Sequestering mTORC1

**FIGURE 3:**
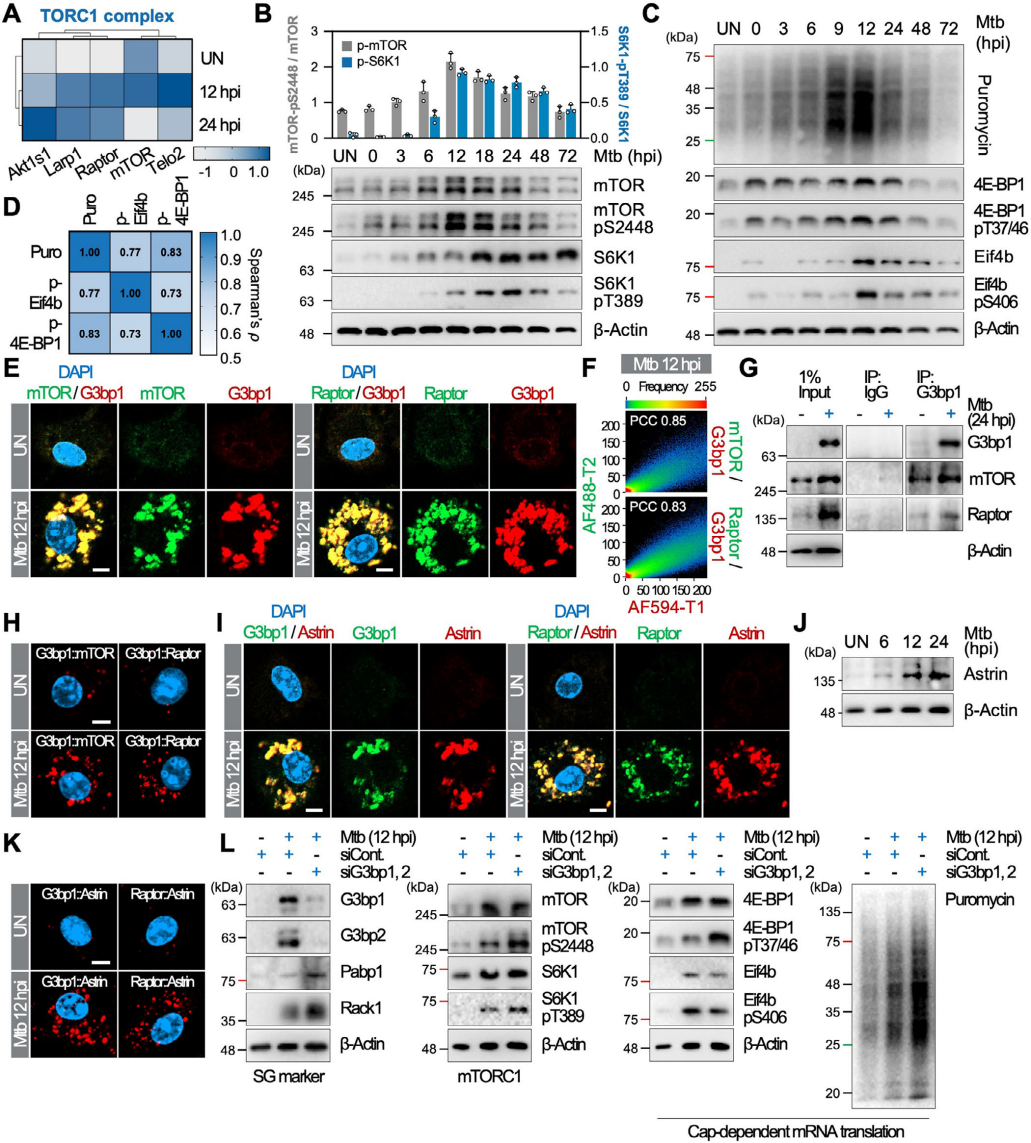
SGs sequester mTORC1 and inhibit cap‐dependent translation during infection. (A) Heatmap of TORC1 complex protein abundance in Mtb‐infected BMDMs (GO: 0031931; MGI v6.22). (B) Immunoblots of mTORC1 activity over time in infected BMDMs (MOI 1); bar graphs show phospho/total ratios. *N* = 3. (C) Immunoblots of cap‐dependent translation‐related proteins in infected BMDMs (MOI 1). (D) Heatmap of Spearman correlation coefficients (*ρ*) for pairwise expression patterns of proteins analyzed in (C). (E) Immunofluorescence showing colocalization of mTOR and Raptor with G3bp1 in infected macrophages (MOI 1, 12 hpi). Scale bar indicates 5 µm. (F) Dual‐color pixel analysis quantifying colocalization of mTORC1 components with G3bp1 from (E). Pearson correlation coefficients (*r*) are shown. (G) Co‐immunoprecipitation of G3bp1 with mTOR and Raptor from infected BMDMs (MOI 1, 24 hpi). (H) PLA showing spatial interaction of mTOR or Raptor with G3bp1 in infected BMDMs (MOI 1, 12 hpi). Scale bar indicates 5 µm. (I) Immunofluorescence of Astrin colocalizing with G3bp1 and Raptor in infected BMDMs (MOI 1, 12 hpi). Scale bar indicates 4 µm. (J) Immunoblot of Astrin expression in infected BMDMs (MOI 1). (K) PLA showing Astrin interactions with G3bp1 and Raptor in infected BMDMs (MOI 1, 12 hpi). Scale bar indicates 5 µm. (L) Immunoblots of SG markers, mTORC1, cap‐dependent translation proteins, and puromycin incorporation in SG^neg^ BMDMs (MOI 1, 12 hpi).

Given that mTORC1 controls cap‐dependent mRNA translation for many mRNAs [34], we examined whether SGs suppress mRNA translation during Mtb infection through mTORC1. We observed that the expression levels of Raptor, mTOR, and Telo2 increased at 12 hpi but declined at 24 hpi, whereas Akt1s1, a negative regulator of mTOR activity [35], and Larp1, a regulatory protein in mTORC1‐mediated cap‐dependent translation [36], increased at both time points (Figure 3A). Consistent with the mRNA translation profile during Mtb infection (Figure 2B), mTORC1 activity increased to 12 hpi and then decreased (Figure 3B). Phosphorylation of 4E‐BP1 and Eif4b followed a similar pattern, and their levels correlated with translation pattern (Figure 3C,D; 4E‐BP1‐pT37/46 vs. Puro, *ρ* = 0.833, *p* = 0.0083; Eif4b‐pS406 vs. Puro, *ρ* = 0.767, *p* = 0.0214).

As mTOR has been identified as a component of SGs under stress conditions [37, 38], we assessed its subcellular localization. During Mtb infection, mTOR and Raptor colocalized with SGs, with a Pearson correlation coefficient value higher than 0.8 (Figure 3E,F; Videos S1 and S2), interacting with G3bp1 (Figure 3G,H). In addition, Astrin, known to recruit Raptor into SGs and suppress mTORC1 [39], was localized within SGs with Raptor (Figure 3I). As mTORC1 activity declined at 12 hpi, Astrin expression increased, and it bound with both G3bp1 and Raptor in Mtb‐infected macrophages (Figure 3J,K; see also Figure 3B,C).

Next, we evaluated the effect of SG on mTORC1 activity and cap‐dependent mRNA translation during Mtb infection. In SG^neg^ macrophages, mTORC1 activity, phosphorylation of translation factors, and puromycin incorporation all increased (Figure 3L). Pabp1 and Rack1, key mediators of cap‐dependent mRNA translation [40, 41], were also increased. Considering their crucial roles in cap‐dependent mRNA translation, the observed increase in these proteins supports enhanced cap‐dependent translation in SG^neg^ macrophages following Mtb infection. Collectively, these findings suggest that Mtb‐induced SGs inhibit cap‐dependent translation by sequestering mTORC1.

### 2.5 Mtb Infection‐Inhibited Autophagy Is Independent of mTORC1

As Mtb‐induced SGs suppress mTORC1, we examined whether SGs affect autophagy via mTORC1. Consistent with prior study [42], lysosome‐associated protein levels decreased during infection, while LC3‐II and p62 accumulated (Figure S3A,B). Notably, autophagy remained inhibited up to 48 hpi, despite decreased mTOR‐pS2448 compared with 12 or 24 hpi (Figures S3B and 3B). This suggests that mTORC1 inhibition does not directly impact autophagy activation in Mtb‐infected macrophages. The cause of reduced lysosomal proteins is still unclear, but ESX‐1‐mediated lysosomal damage [14, 43, 44] and endolysosomal damage likely contribute to the impaired autophagy during Mtb infection (Figure S3B).

To test whether this mTORC1‐autophagy disconnect is specific to Mtb infection, we analyzed p62 turnover during low‐glucose (LG) starvation. Starvation progressively reduced mTORC1 activity and p62 levels, and bafilomycin A1 (Baf. A1) reversed this effect (Figure S3C). In contrast, p62 levels in Mtb‐infected macrophages were unaffected by mTORC1 status (Figure S3D). Furthermore, a comparison of Mtb‐infected cells with autophagy‐inhibited cells revealed that the characteristic structures of autophagy, such as autolysosomes and autophagosomes, remained largely unchanged (Figure S3E,F). Importantly, lysosomal levels were reduced in Mtb‐infected macrophages compared to uninfected bystander cells (Figure S3G), suggesting that lysosomal damage disrupts autophagic flux. These results indicate that Mtb suppress autophagy independently of SG‐mTORC1 signaling. Lysosomal damage appears to be a key factor, possibly, impairing, fusion, and degradation steps of autophagy flux, and mTORC1 may later shift toward other cellular pathways, uncoupling from autophagy regulation.

### 2.6 SGs Inhibit Immune Response‐ and Mitochondrion‐Associated mRNA Translation of Macrophages

**FIGURE 4:**
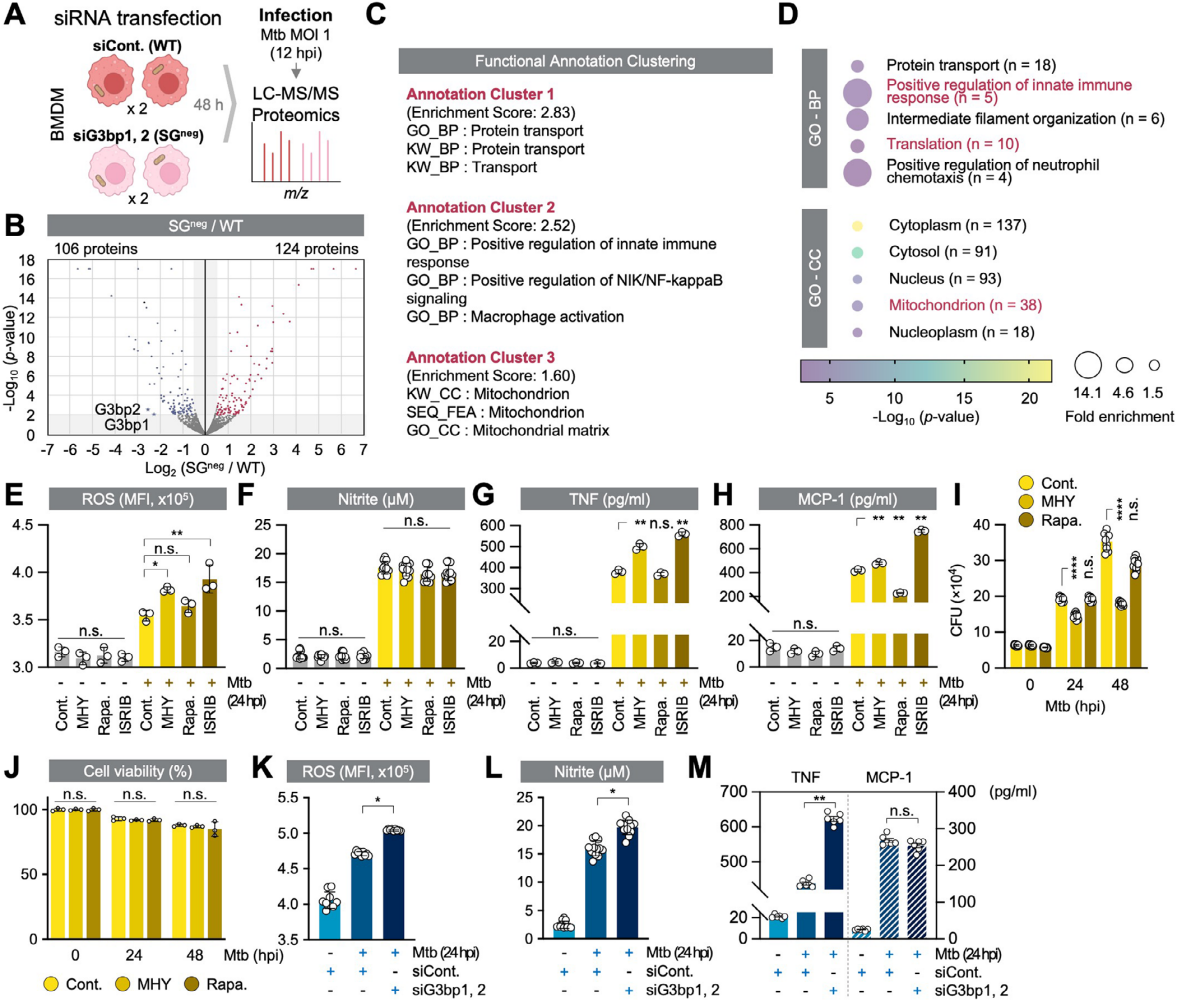
SGs suppress mitochondrial and immune protein translation, impairing macrophage activation. (A) Quantitative proteomics profiling of WT and SG^neg^ BMDMs (MOI 1, 12 hpi, LC–MS/MS). (B) Volcano plot of −log_10_ (*p* value) versus log_2_ FC (dKD/control). A total of 106 proteins with −log_10_ (*p* value) > 2 and log_2_ FC ← 0.5 are colored in blue. A total of 124 proteins with −log_10_ (*p* value) > 2 and log_2_ FC > +0.5 are colored in red. (C) Functional annotation of 124 proteins elevated in SG^neg^ macrophages from (B). Top three clusters are shown based on enrichment scores. (D) Bubble plot of GO terms (biological process (BP) and cellular component (CC)) for the 124 proteins. (E, F) ROS (E; *N* = 3) and nitrite (F; *N* = 10) production was measured in media of BMDMs treated with MHY1485 (MHY), rapamycin (Rapa.), or ISRIB, then infected (MOI 1, 24 hpi). n.s., nonsignificant, *p < 0.05, **p < 0.01. (G, H) TNF (G; *N* = 3) and MCP‐1 (H; *N* = 3) production was measured in media of BMDMs treated with MHY, Rapa., or ISRIB, then infected (MOI 1, 24 hpi). n.s., nonsignificant, **p < 0.01. (I) Intracellular survival of Mtb in BMDMs ± MHY or Rapa. (MOI 1). Data from three independent experiments are shown as mean ± SEM. n.s., nonsignificant, ****p < 0.0001. *N* = 9. (J) Cell viability (LDH assay) after MHY or Rapa. ± infection (MOI 1). Relative to 0 hpi control (Cont.). n.s., nonsignificant; compared within the same time point. *N* = 3. (K, L) ROS (K; *N* = 9) and nitrite (L; *N* = 12) production was measured in media of WT or SG^neg^ BMDMs (MOI 1, 24 hpi). *p < 0.05. (M) TNF (*N* = 6) and MCP‐1 (*N* = 6) production was measured in media of WT and SG^neg^ BMDMs (MOI 1, 24 hpi). n.s., nonsignificant, **p < 0.01.

As enhanced mRNA translation restricted Mtb growth (Figure 2G) and Mtb‐induced SGs inhibited mTORC1 and cap‐dependent translation (Figure 3L), we hypothesized that SGs block host mRNA translation to help Mtb survival. To examine the biological processes regulated by SGs, we compared the proteomes of Mtb‐infected wild type (WT) and SG^neg^ macrophages infected with Mtb (Figure 4A). We identified 230 differentially expressed proteins (Figure 4B and Table S2); 124 proteins were increased in SG^neg^ macrophages and enriched for protein transport, innate immune response, macrophage activation, and mitochondria (Figure 4C,D). Importantly, SG deficiency did not induce compensatory activation of alternative stress pathways, instead, the expression of key stress‐related genes was reduced, suggesting that SGs might act as amplifiers of stress signaling rather than passive markers of cellular stress (Figure S4A–C). Together, these data suggest that Mtb promotes its survival by sequestering mTORC1 in SGs, thereby suppressing translation of mRNAs involved in innate immune defense and mitochondrial activity.

### 2.7 SGs Regulate ROS, NO, and Cytokine‐Mediated Innate Immunity During Mtb Infection

Given the enrichment of innate immune and mitochondrial proteins in SG^neg^ macrophages (Figure 4C), we examined whether the absence of SGs or pharmacologic mTORC1 activation restores microbicidal activity of macrophages. Activation of mTORC1 with MHY1485 or enhancement of translation with ISRIB increased ROS production upon Mtb infection, while nitrite (NO) production remained unchanged (Figure 4E,F). Both treatments also increased the production of tumor necrosis factor (TNF) and monocyte chemoattractant protein‐1 (MCP‐1; Figure 4G,H). Consistent with the roles of ROS, NO, TNF, and MCP‐1 in controlling Mtb [45, 46, 47], intracellular Mtb survival decreased in mTOR‐activated and ISRIB‐treated macrophages without affecting host cell viability (Figure 4I,J; see also Figure 2G,H). ROS, NO, and TNF were significantly increased in SG^neg^ macrophages, while MCP‐1 remained stable (Figure 4K–M), reflecting their enhanced anti‐Mtb activity (Figure 2O,P).

However, as those inhibitory effects of siRNA‐mediated dKD of G3bp1 and 2 could be specific roles of G3bp proteins and not Mtb‐induced SGs per se, we confirmed using uninfected BMDMs with no SGs, transfected with siG3bp1 and 2 (Figure S5A). In the absence of Mtb infection, dKD did not alter translation, mTORC1 activity, cap‐dependent translation‐associated protein expression levels or basal innate immunity (Figure S5B–G). Collectively, these observations indicate that SGs suppress key effector mechanisms, including ROS, NO, and cytokine production, against Mtb infection.

### 2.8 SGs Regulate Mitochondrial Activity Upon Mtb Infection

**FIGURE 5:**
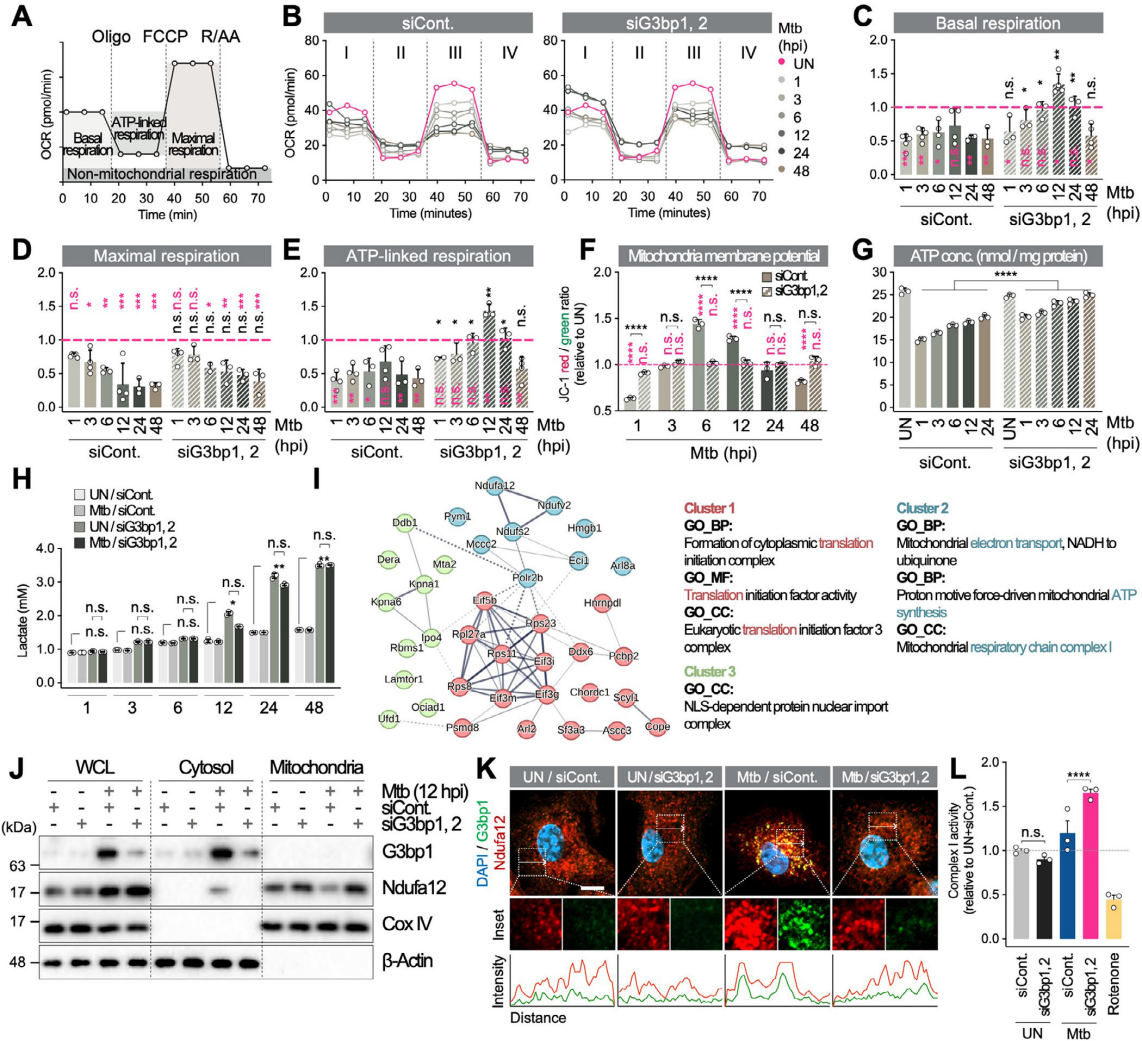
SGs impair mitochondrial complex I activity during Mtb infection. (A) Scheme illustrating the breakdown of oxygen consumption following the addition of the specified inhibitors for assessing oxygen consumption rates (OCRs). (B) OCRs in WT and SG^neg^ macrophages infected with Mtb (MOI 1). Cells were transfected with either siControl (left) or siG3bp1/2 (right) for 48 h before infection. (C–E) Quantification of relative basal (C), maximal (D), and ATP‐linked respirations (E) from (B). Normalized to each UN control (pink‐colored dotted line). Pink *p* value versus UN, black *p* value versus siCont. n.s., nonsignificant, *p < 0.05, **p < 0.01. *N* = 3. (F) Mitochondrial membrane potential (JC‐1 red/green ratio) in infected WT and SG^neg^ BMDMs (MOI 1; normalized to UN; pink dotted line). n.s., nonsignificant, ****p < 0.0001. *N* = 3. (G) Intracellular ATP concentrations in infected WT and SG^neg^ BMDMs (MOI 1). ****p < 0.0001. *N* = 3. (H) Lactate secretion from UN or Mtb‐infected WT and SG^neg^ BMDMs (MOI 1). n.s., nonsignificant, *p < 0.05, **p < 0.01. *N* = 3. (I) STRING analysis (v11.5) of 37 proteins upregulated in SG^neg^ BMDMs and simultaneously present in the SG proteome, grouped into translation (Cluster 1), mitochondria (Cluster 2), and nuclear import complex (Cluster 3). (J) Immunoblot of whole‐cell lysate (WCL), cytosolic fraction, and mitochondria fraction from Mtb‐infected WT and SG^neg^ BMDMs (MOI 1, 12 hpi). (K) Immunofluorescence analysis of G3bp1‐Ndufa12 colocalization in Mtb‐infected WT and SG^neg^ BMDMs (MOI 1, 12 hpi); line profiles show fluorescence intensity (white arrow). Scale bar indicates 5 µm. (L) Mitochondrial complex I activity in Mtb‐infected WT and SG^neg^ BMDMs (MOI 1, 12 hpi); rotenone as positive control. Relative mitochondrial complex I activity is plotted, compared to uninfected siControl cells. n.s., nonsignificant, ****p < 0.0001. *N* = 3.

A notable observation is the significant recovery of mitochondria‐associated proteins (cluster 3) in SG^neg^ macrophages (Figure 4C,D). The proteins comprising this cluster are implicated in mitochondrial function, and their recovery indicates a plausible involvement in enhancing mitochondrial activity. We therefore hypothesized that SGs impair macrophage activity by limiting mitochondrial function, thereby favoring Mtb survival. To test this, we compared mitochondrial function in WT and SG^neg^ macrophages during Mtb infection (Figure 5A,B). Basal respiration, reduced in Mtb‐infected cell versus UN, was significantly restored in SG^neg^ macrophages (Figure 5C). Maximal respiration remained below UN levels but showed no significant difference between WT and SG^neg^ macrophages (Figure 5D). ATP‐linked respiration was higher in SG^neg^ macrophages (Figure 5E). Mitochondrial membrane potential (MMP) fluctuated over time in WT but remained stable in SG^neg^ macrophages, suggesting that SGs affect mitochondrial homeostasis (Figure 5F). Accordingly, SG^neg^ macrophages exhibited significantly increased ATP levels (Figure 5G) consistent with greater ATP‐linked respiration and suggesting mitochondria, not glycolysis, as the primary source (Figure 5H).

### 2.9 SGs Inhibit Mitochondrial Complex I Activity by Sequestering Ndufa12

To explore how SGs regulate mitochondrial function, we analyzed 37 proteins from those recovered in Mtb‐infected SG^neg^ macrophages that overlap with the known SG proteome (Figure 5I) [38, 48]. These 37 proteins were grouped into three clusters: translation, mitochondrial, and nuclear import (Figure 5I). We found that Ndufa12, Ndufv2, and Ndufs2, which are components of mitochondrial complex I, were included in the SG proteome as well as increased in SG^neg^ macrophages (Figure 5I and Table S3). Because complex I initiates electron transfer to ubiquinone and generates the proton gradient for ATP synthesis [49], we focused on Ndufa12, which showed the largest increase.

Mtb infection elevated Ndufa12 expression, with a further increase in SG^neg^ macrophages (Figure 5J). In WT macrophages, Ndufa12 partly relocated from mitochondria to SG‐containing cytosol, whereas in SG^neg^ macrophages, it remained in mitochondria (Figure 5J,K). To assess the functional consequences of Ndufa12 translocation from mitochondria to SGs, we measured complex I activity in mitochondria. The activity was significantly increased in SG^neg^ macrophages (Figure 5L), indicating that Mtb‐induced SGs impair mitochondrial complex I by sequestering Ndufa12.

### 2.10 SG Formation in Mtb‐Infected Macrophages Occurs Independently of Lysosomal Damage or Oxidative Stress

We next investigated factors initiating SG formation during Mtb infection. Although lysosomal damage via the ESX‐1 system promotes SG assembly [14, 15], SGs also formed in macrophages infected with the avirulent strain H37Ra (ESX‐1 impaired) or bacillus Calmette–Guérin (BCG; lacking ESX‐1; Figure S6A). H37Rv infection produced more SGs per cell than H37Ra or BCG, yet the fraction of SG^+^ cells was similar (Figure S6B,C), and phagosomal damage did not differ between WT and SG^neg^ cells (Figure S6D,E). This finding indicates that lysosomal damage may not be a primary contributing factor to SG formation during Mtb infection, despite the significant lysosomal damage induced by the infection.

It is noteworthy that increasing MOI tended to raise the ratio of SG^+^ cells upon H37Rv infection (Figure S6F,G). These observations imply a correlation between the number of Mtb infecting macrophages and the subsequent formation of SGs. This phenomenon can be explained by two hypotheses: either there is an increase in the number of Mtb being phagocytosed, or Mtb induces a more pronounced stress response within the cells. To test this, we first examined whether the stress response induced by Mtb influences the formation of SGs. As previous studies have shown that Mtb infection induces oxidative stress [50, 51], we investigated how SG formation was affected when cells were treated with ROS scavengers. The results demonstrated that infection‐induced SGs remained unaffected by oxidative stress (Figure S6H–J).

### 2.11 Intracellular ATP Levels Control Infection‐Induced SG Assembly and Disassembly and Mtb Survival

**FIGURE 6:**
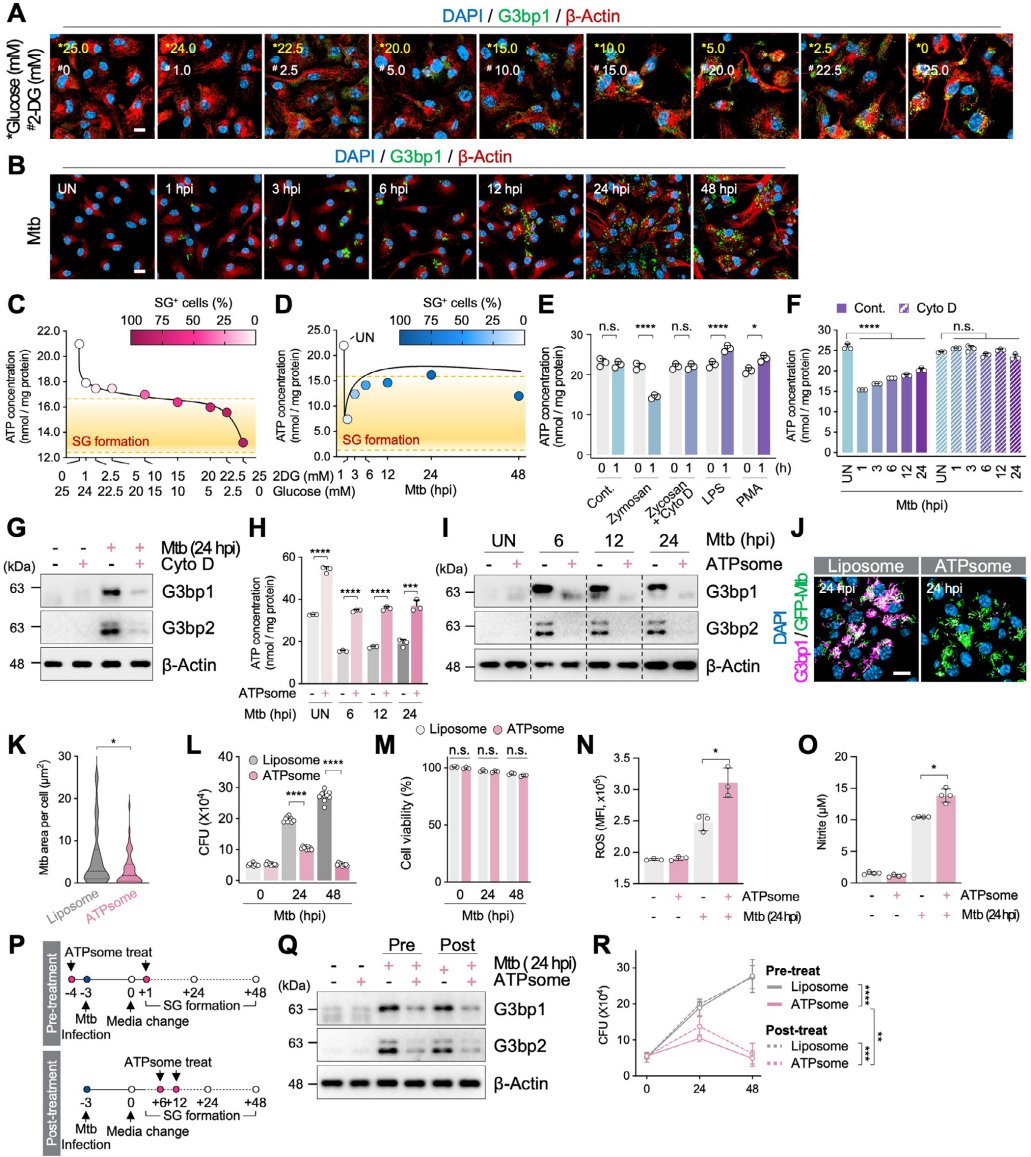
Intracellular ATP level regulates the dynamics of SGs which affect innate immune activity of macrophages. (A) Immunofluorescence analysis of SGs BMDMs treated with 2‐DG or glucose depletion for 24 h. Scale bar indicates 10 µm. (B) Immunofluorescence analysis of SGs in BMDMs infected with Mtb (MOI 1). Scale bar indicates 10 µm. (C) Intracellular ATP concentrations in BMDMs from (A) were plotted with SG‐positive ratio. The ATP concentration (17.5 nmol/mg protein) correlating with 50% of SG‐positive cells is indicated with dotted line and considered as SG‐inducing ATP concentration. (D) Intracellular ATP concentrations of BMDMs from (B) were plotted with SG‐positive ratio. The SG‐inducing ATP concentration (17.5 nmol/mg protein) from (C) is indicated with dotted line. (E) Intracellular ATP concentrations in zymosan, LPS, and PMA‐treated BMDMs. n.s., nonsignificant, *p < 0.05, ****p < 0.0001. *N* = 3. (F) Intracellular ATP concentrations in Mtb‐infected BMDMs, following cytochalasin D (Cyto D) treatment. n.s., nonsignificant, ****p < 0.0001. *N* = 3. (G) Immunoblot analysis of SG markers in BMDMs ± Cyto D after Mtb infection (MOI 1, 24 hpi). (H) Intracellular ATP concentrations in BMDMs treated with empty‐liposome or ATPsome before Mtb infection (MOI 1). n.s., nonsignificant, ***p < 0.001, ****p < 0.0001. *N* = 3. (I) Immunoblot analysis of SG markers in BMDMs treated as in (H). (J) Immunofluorescence analysis of SGs in GFP‐Mtb‐infected BMDMs ± ATPsome. Scale bar indicates 10 µm. (K) Quantification of Mtb area per cell in (J). *p < 0.05. *N* = 15. (L) Intracellular Mtb survival in BMDMs ± ATPsome. Data from three independent experiments are shown as mean ± SEM. ****p < 0.0001. *N* = 3. (M) BMDM viability (LDH assay) after ATPsome or liposome treatment ± infection (MOI 1) Relative to 0 hpi control (Liposome). n.s., nonsignificant; compared within the same time point. *N* = 3. (N, O) ROS (N; *N* = 3) and nitrite (O; *N* = 3) production was measured in media of BMDMs (MOI 1, 24 hpi) ± ATPsome. *p < 0.05. (P) Experimental scheme for pre‐ or post‐treatment of ATPsome. (Q) Immunoblot analysis of BMDMs pre‐ or post‐treated with ATPsome as in (P) (MOI 1, 24 hpi). (R) Intracellular Mtb survival in BMDMs pre/post‐ATPsome treatments (MOI 1). Data from three independent experiments are shown as mean ± SEM. n.s., nonsignificant, **p < 0.01, ***p < 0.001, ****p < 0.0001. *N* = 9.

Interestingly, intracellular ATP concentration was rapidly decreased after Mtb infection (Figure 5G). Given that intracellular energy deficiency induces SG formation [26], we examined whether ATP deficiency initiates SG formation. Treating macrophages with 2‐deoxy‐D‐glucose (2‐DG) and lowering glucose showed that SGs formed when ATP fell below ∼17.5 nmol (Figure 6A,C). Accordingly, we considered 17.5 nmol ATP as the initial concentration for SG formation. Next, intracellular ATP levels were measured after Mtb infection. At 1 hpi, ATP concentration dropped to 7.5 nmol, which was much lower than the initial concentration required for SG formation (Figure 6B,D). The rapid reduction in ATP levels was thought to be due to phagocytosis. This idea is further supported by several papers showing ATP consumption during phagocytosis [52, 53, 54].

To test this hypothesis under Mtb‐infected macrophages, we tested whether phagocytosis directly affect intracellular ATP levels or whether other immune signals lead to ATP decrease. Zymosan, a phagocytic particle [55], reduced ATP within 1 h, whereas cytochalasin D (Cyto D) prevented this reduction; LPS (TLR4 activator) or phorbol 12‐myristate 13‐acetate (PMA; NF‐ĸB inducer) slightly increased intracellular ATP levels (Figure 6E). These results suggest that intracellular ATP reduction is driven by phagocytosis rather than immune signaling cascades associated with Mtb infection. Next, we examined how intracellular ATP level change over time during Mtb infection with phagocytosis inhibited. Macrophages undergoing phagocytosis showed a rapid ATP drop after infection, whereas ATP remained stable when phagocytosis was blocked (Figure 6F). SGs were absent in Cyto D‐treated cells (Figure 6G), indicating that phagocytosis‐driven ATP depletion triggers SG assembly.

In order to examine the effect of ATP on SG formation, we used ATP‐containing liposomes (ATPsomes) to restore ATP depletion during Mtb infection. ATPsomes increased ATP to UN levels (Figure 6H) and markedly reduced SG formation (Figure 6I,J).

To confirm the relationship between ATP levels and SG dynamics, we modulated ATP with rotenone and antimycin A (Rot/AA; depletion) or ATPsomes (replenishment) in both uninfected and Mtb‐infected BMDMs (Figure S7A,B). SG numbers inversely correlated with ATP levels, supporting direct energy‐dependent SG regulation (Figure S7C,D). Consistently, replenishing ATP reduced Mtb survival without affecting viability (Figure 6K–M) and increased ROS and NO production (Figure 6N,O). Finally, ATPsomes disassembled preformed SGs and further lowered Mtb burden (Figure 6P–R). These results demonstrated that severe ATP depletion by Mtb infection induced SG formation and those SGs were implicated in the innate immune activity of macrophages and TB pathogenesis.

### 2.12 SGs Inhibit the Cellular Activity of Macrophages Upon *Listeria monocytogenes* Infection

To determine whether other intracellular bacteria also trigger SG formation and suppress host immunity, we examined *Listeria monocytogenes* (Lm), which causes listeriosis and evades host defense [56]. Lm infection induced SG formation via phagocytosis‐driven ATP depletion (Figure S8A–C). As with Mtb, Lm infection transiently increased mTORC1 activity and cap‐dependent translation, which decreased by 6 hpi (Figure S8D). Upon Lm infection, SG^neg^ macrophages showed higher mTORC1 activity and cap‐dependent translation (Figure S8E) and produced more ROS, NO, and TNF, whereas MCP‐1 remained unchanged (Figure S8F–I; see also Figure 4M). Correspondingly, intracellular Lm survival was significantly suppressed in SG^neg^ macrophages (Figure S8J). These results demonstrate that SGs induced by pathogenic intracellular bacterial infections, not confined to Mtb, help the survival of bacteria by inhibiting host cellular activities, such as mRNA translation, energy homeostasis, and innate immune responses.

### 2.13 SGs Impair Host Defense Against Mtb In Vivo by Suppressing mTORC1 and Cytokine Production

**FIGURE 7:**
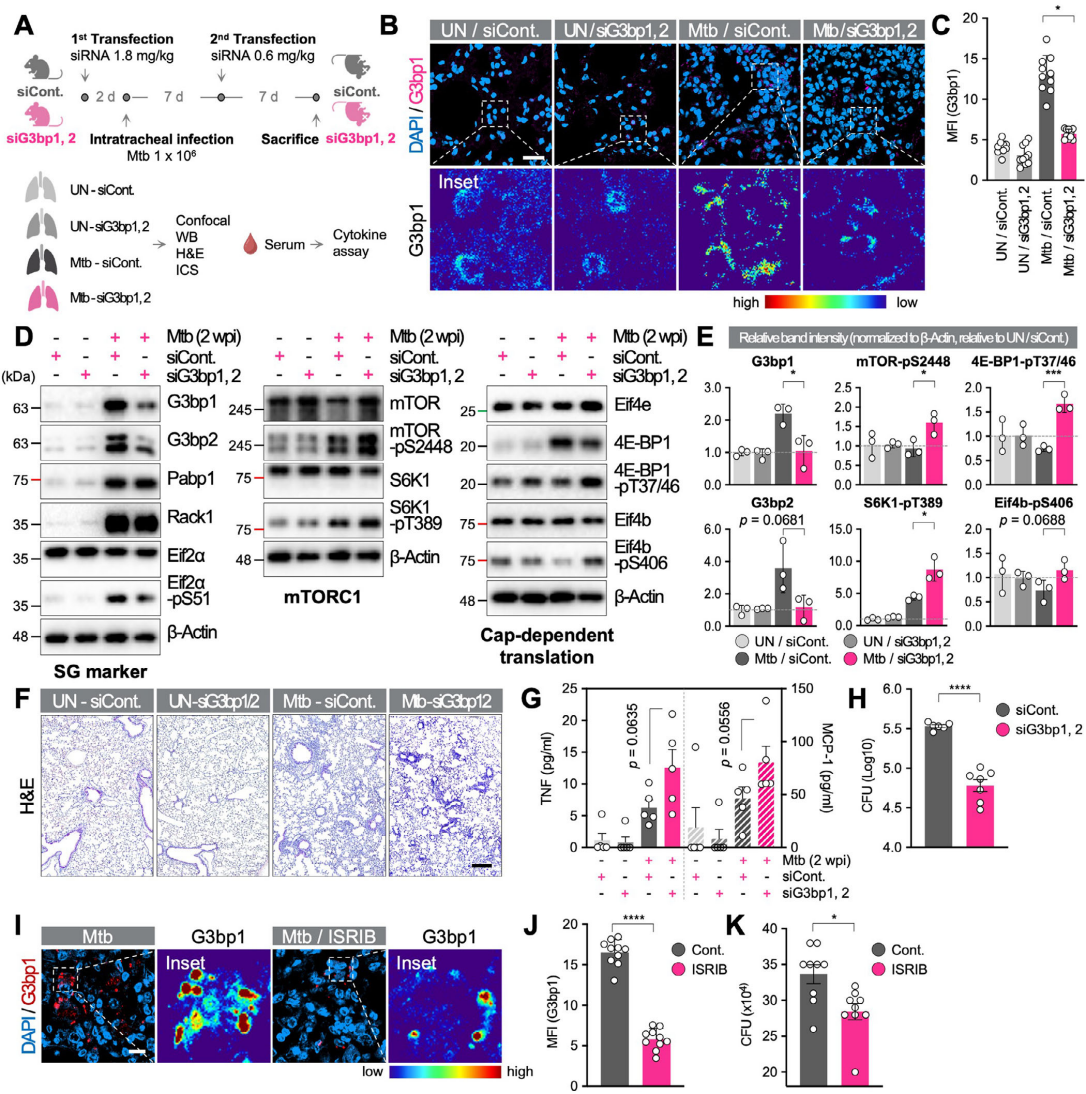
Inhibition of SGs restores macrophage immune function and restricts Mtb in vivo. (A) Experimental scheme for the in vivo experiments. Mice were transfected with siRNAs via intravenous (i.v.) injection, followed by intratracheal infection with Mtb for 7 days. Seven days postinfection, siRNA transfection was repeated, and the mice were sacrificed another 7 days later for experiments. (B) Immunofluorescence analysis of lung tissues from siRNA‐transfected uninfected (UN) or Mtb‐infected mice. G3bp1 fluorescence intensity was visualized in rainbow color. Scale bar indicates 20 µm. (C) Mean fluorescence intensity (MFI) of G3bp1 signals from (B). *p < 0.05. *N* = 10. (D) Immunoblot analysis of SG markers, mTORC1, and cap‐dependent translation proteins in lung lysates. (E) Quantification of relative band intensities from (D). Data are presented as relative band intensities compared to uninfected siControl group (UN/siCont.). *p < 0.05, ***p < 0.001. *N* = 3. (F) H&E staining of lung tissues from siRNA‐transfected uninfected (UN) or Mtb‐infected mice. Scale bar indicates 200 µm. (G) Quantification of TNF and MCP‐1 levels in mouse serum. Data are presented as mean ± SEM. *N* = 5. (H) Lung CFU in Mtb‐infected mouse lung tissues. Data are presented as mean ± SEM. ****p < 0.0001. *N* = 7. (I) Immunofluorescence analysis of lung tissues from ISRIB‐treated and Mtb‐infected mice. G3bp1 fluorescence intensity was visualized in rainbow color. Scale bar indicates 10 µm. (J) MFI of G3bp1 from (H). ****p < 0.0001. *N* = 10. (K) Lung CFU in ISRIB‐treated and Mtb‐infected mouse lung tissues. Data are presented as mean ± SEM. *p < 0.05. *N* = 9.

Finally, we evaluated SG effects on host defense in Mtb‐infected mice (Figure 7A). Lungs of SG^neg^ mice displayed markedly fewer SGs after Mtb infection (Figure 7B,C). Consistent with our in vitro findings, we observed an increase in mTORC1 activity and cap‐dependent translation‐related proteins in SG^neg^ mice (Figure 7D,E). Histopathological analysis further revealed stronger inflammatory infiltrates in lungs of SG^neg^ mice compared with WT mice (Figure 7F). In addition, the cytokine productions were significantly increased in SG^neg^ mice serum than that of WT mice (Figure 7G). These effects on innate immune activity ultimately resulted in a significant decrease in Mtb survival in the lung tissues of SG^neg^ mice (Figure 7H). Consistently, pharmacological inhibition of SG formation with ISRIB treatment also led to a marked reduction in SGs in lung tissues (Figure 7I,J) and decreased Mtb burdens, confirming the role of SG disruption in enhancing host defense against Mtb (Figure 7K). Overall, this study elucidates the previously unknown role of SGs in Mtb infection by demonstrating that SGs act as an integrated negative regulator of cellular activity, including mRNA translation, energy homeostasis, and innate immune response of macrophages against Mtb infection.

## 3 Discussion

Consistent with the importance of immunometabolism in immune cell activity [57], TB pathogenesis is associated with the disruption of immunometabolism in various immune cells [58]. This compromised metabolic homeostasis has been attributed to the development of TB [59]. In this study, we hypothesized that an energy imbalance may contribute to the pathogenesis of TB. This speculation is supported by our results showing that energy depletion‐induced SGs downregulate immune‐ and mitochondria‐related proteins over a longer duration of Mtb infection. Mtb infection‐induced SGs have three distinct features related to cellular activity: duration of infection, regulation of intracellular energy homeostasis, and the innate immune response.

First, in terms of the duration of Mtb infection, we observed that mRNA translation and mTORC1 activity decreased and that the cellular proteome and mitochondrial activity were altered when SGs were saturated. Considering that the duration of infection may govern cellular activities regulated by ATP‐mediated SGs, the idea that differences in TB development may be driven by the duration of infection and cellular stress in individuals is further supported. Second, in terms of intracellular energy homeostasis, our results showing increased mTORC1 activity, cap‐dependent translation, and mitochondrial activity in SG^neg^ cells support the hypothesis that mTORC1 increases mitochondrial activity and biogenesis through mRNA translation [60]. Given that ATP recovery inhibits SG formation and disassembles SGs, our study shows that SG‐regulated energy homeostasis may be an important factor in determining the development and symptoms of TB. Third, the innate immune response is an integrated effect of the two aforementioned SG features. We believe that the decrease in immune‐ and mitochondria‐related proteins due to SG formation may adversely affect innate immune activity in Mtb‐infected macrophages, contributing to TB pathogenesis. Our proteomic data further demonstrated that the absence of SGs leads to increased expression of mitochondrial‐ and immune‐related proteins (Figure 4C,D), and these changes are functional consequences directly reflected by the proteomic dataset, as evidenced by enhanced OXPHOS, ROS, NO, and cytokine production (Figures 4K–M and 5C). These findings support our mechanistic model in which SG‐mediated suppression of mTORC1 activity and mRNA translation leads to reduced energy metabolism and innate immune effector responses. Collectively, energy status, which affects the regulation of SG formation, may determine the pathogenesis of TB.

It has been demonstrated that certain viruses induce SG formation to facilitate their replication, thereby shifting the role of SGs from host protection to viral benefit [61]. Conversely, we hypothesize that SGs induced by Mtb infection serve a Mtb‐beneficial role. In the initial phases of Mtb infection (up to 12 hpi), Eif2α undergoes phosphorylation (as part of the ISR) and mTORC1 activity is elevated, leading to augmented cap‐dependent translation (Figure 3B,C). This is presumably essential for sustaining energy supply and protein synthesis, which are crucial for mounting an effective immune response. However, SG formation also increases during this period, reaching a peak at 12 hpi (Figures 1G and 6B), where excessive SG accumulation suppresses mTORC1 activity, mitochondrial function, and cap‐dependent translation. This suggests that while initial mTORC1 activation supports macrophage defense mechanisms, prolonged SG formation (potentially by sustained ISR; phosphorylated Eif2α) disrupts integrated cellular activities, including energy production and protein synthesis, ultimately impairing the macrophage's ability to control Mtb. Consequently, in the context of prolonged infection with sustained ISR and SG formation, SG disassembly could be advantageous for macrophages by restoring cellular homeostasis and enhancing their capacity to combat Mtb. The present findings are consistent with those of studies emphasizing the role of the ISR in TB susceptibility and control. Notably, recent research has demonstrated that ISRIB improves outcomes in mouse TB models by mitigating the detrimental effects of sustained ISR activation [62, 63, 64].

The formation of SGs is generally regarded as an adaptive response to cellular stress, with the primary function of preserving cellular homeostasis and promoting survival under stress conditions. However, the present study reveals a paradoxical effect of SGs, wherein they facilitate the survival of pathogens (Mtb and Lm), thereby raising the question of why macrophages induce SGs that are ultimately detrimental to host cells during the infection. One potential explanation for this phenomenon is that the formation of SGs in response to these infections is an evolutionary conserved adaptive mechanism of cells triggered by various cellular stress responses, including kinase activation, ATP depletion, and translational stalling. While SGs are generally beneficial, such as during viral infections or noninfectious cellular stress (e.g., oxidative stress), they can become maladaptive in specific cases of Mtb and Lm infections. This is due to the ability of these pathogens to exploit SGs to support their own survival and replication in macrophages. Alternatively, SG formation may represent an imperfect or incomplete response by macrophages to these pathogens. These pathogens may have evolved to have strategies to utilize SGs or to thrive within the SG‐mediated environment, converting a host survival mechanism to their advantage. Future studies should focus on understanding why macrophages form SGs that are harmful to host cells, revealing the evolutionary and molecular mechanisms that drive this paradoxical response during Mtb and Lm infections.

A recent study has proposed an alternative model of SGs, in which SGs are formed by the ESX‐1‐positive Mtb infection. This model suggests that SGs can restore lysosomal damage caused by the ESX‐1 system, thereby decreasing the Mtb area per cell [15]. Another study also demonstrated the relationship between lysosomal damage and SGs, indicating that lysosomal damage‐induced SGs interacted with lysosomes and suppressed mTORC1 activity [14]. In contrast, the present study suggests that SG assembly is not primarily driven by lysosomal damage. We observed significant SG formation in the presence of ESX‐1‐impaired or ‐lacking Mtb strains, which exhibit reduced capacity to induce lysosomal damage (Figure S6A). Furthermore, the supplementation of ATP prior to infection effectively impeded SG formation, despite the potential for persistent lysosomal damage resulting from Mtb infection (Figure 6I,J). However, we also noted a trend toward increased SG numbers per cell during H37Rv infection compared to BCG and H37Ra infections (Figure S6B). Consequently, we propose the hypothesis that ATP depletion induced by Mtb infection is the predominant factor driving the process of SG assembly, while lysosomal damage contributes partially to this process.

The observed discrepancies are likely due to differences in the experimental methods used to assess Mtb survival within macrophages. Specifically, their study utilized fluorescence‐labeled Mtb to calculate the bacterial area per cell [15]; however, this approach has limitations in distinguishing between live and dead Mtb. Furthermore, it is unable to differentiate between Mtb that is engulfed within the cell and Mtb attached to the cell membrane. The tendency of Mtb to form clusters and infect macrophages unevenly suggests that measuring the Mtb area per cell may not accurately reflect the anti‐Mtb effects of SGs, given the significant variation in the number of bacteria per macrophage. From a pathological perspective, accurately measuring only live Mtb is essential to evaluate the impact of SGs on Mtb control within macrophages. Nevertheless, macrophages treated with ATPsome exhibited a consistent trend of reduced Mtb area per cell, accompanied by a notable decrease in CFU (Figure 6K,L).

Consistent with the proposed function of SGs induced by Mtb in our study, we also found that Lm infection also induced ATP depletion and SG formation, resulting in the inhibition of mTORC1 translation and innate immune responses. These results are particularly important, suggesting that the regulatory role of SGs over macrophage antimicrobial activity applies not only to Mtb infection but also to other intracellular bacterial infections. Various immune evasion mechanisms of intracellular pathogens, including the modulation of immunometabolism and phagosomal maturation, may explain their survival within host cells [65]; however, these mechanisms cannot account for differences in disease development and symptoms among infected individuals. Our results demonstrating energy‐ and stress status‐mediated alterations in cellular activity provide evidence for differences in TB development among infected individuals.

Several studies have focused on the mechanistic role of SGs in mRNA translation [32]; however, it remains controversial whether SGs can inhibit mRNA translation [66, 67]. We found that mRNA translation gradually increased early during infection, when the number of SGs was low, whereas upon the saturation of SG formation, mRNA translation significantly decreased. Our findings imply that mRNA translation is inhibited in the presence of SGs above a certain level and that these mRNAs may be restricted to mTOR‐dependent translation and are important for mitochondrial activity and innate immune responses, at least for Mtb and Lm infections.

SG^neg^ cells showed increased ROS, NO, and TNF, whereas MCP‐1 was unaffected. In addition, mTORC1‐activated and ISRIB‐treated cells showed increased levels of ROS, TNF, and MCP‐1 but no significant difference in NO levels. These differences suggest that SGs have regulatory functions in the innate immune response, including the activation of mTORC1. Importantly, the modulation of mTORC1 activity affects autophagy. Given the implications of xenophagy for invading pathogens [68], it is important to consider how mTORC1 activation might inhibit intracellular mycobacterial survival. Although the activation of autophagy is important for innate defense against Mtb, our results show a decrease in lysosome‐associated proteins and an accumulation of p62 and LC3‐II in Mtb‐infected cells, suggesting a different regulatory mechanism for autophagy in this condition. We hypothesize that mTORC1 activation in the absence of SGs, or the pharmacological activation of mTORC1, may not affect autophagy because autophagy is already suppressed in Mtb‐infected macrophages. Therefore, we propose that, in addition to activating autophagy to eliminate bacteria, increasing the expression of mitochondrial‐ and immune‐related proteins, along with enhancing global mRNA translation, may have a more integrative impact on controlling Mtb within macrophages, thereby promoting innate immune responses.

A notable finding is the observation that type I IFN response is enhanced by SGs [18]. The progression of TB in patients has been shown to be closely associated with hyperactivation of the type I IFN response [69, 70, 71]. Furthermore, studies using mouse TB models and human monocyte‐derived macrophages have demonstrated that type I IFN response plays a critical role in TB pathogenesis by disrupting Mtb control and increasing susceptibility to Mtb [19, 72, 73, 74]. Most recently, type I IFN‐mediated neutrophil extracellular traps have been shown to promote Mtb replication and tissue damage [75]. These findings suggest that SGs, which are induced by Mtb infection, contribute to increased type I IFN response, thereby impairing macrophage‐mediated TB control and influencing disease progression. This supports the hypothesis that Mtb‐induced SGs help the intracellular survival of Mtb by reducing macrophage activity.

Notably, the inhibition of SG formation had a greater impact on antimycobacterial activity in vivo than in vitro (decrease of 39.8% in vitro and 49.4% in vivo). This suggests that siRNA‐mediated knockdown of G3bp1/2 also affects other immune cells and that the interactions of immune cells have a synergistic effect on enhancing the immune activity of macrophages against Mtb infection. Studies showing an increased type I IFN response by SGs [18] and impaired macrophage control of Mtb by type I IFN in neutrophils [19] support our findings.

In our experimental design, we did not distinguish between infected and uninfected bystander cells, instead focusing on the collective and integrated cellular responses to Mtb infection. Given that approximately 60% of infected cells and 20% of uninfected bystander cells form SGs, the observed SG‐mediated phenotypes likely reflect dominant mechanisms during infection. Nevertheless, resolving these populations at the single‐cell level could uncover additional heterogeneity and context‐specific responses. Future studies investigating the subcellular localization and translational control of specific immune transcripts will be essential to dissect the gene‐specific regulatory roles of SGs beyond their global effects on translation. Furthermore, the exact composition of the proteins in Mtb‐induced SGs is still unclear. Previous studies have shown that some pathogens remodel SGs, generating atypical condensates with distinct protein profiles [76, 77]. Therefore, it is plausible that Mtb repurposes SGs in a similar manner via its own proteins. Future work should define the proteomic profile of these SGs and its implications for host–pathogen interactions.

In conclusion, our results demonstrate that SGs induced by intracellular energy depletion during Mtb infection play an important role in the immunometabolic events that regulate Mtb survival within macrophages. We propose a comprehensive biological and physiological role for SGs in Mtb infection that integrates mRNA translation, mTORC1, and metabolic and immune regulation. We believe that our proposed mechanism of SG‐mediated dysregulation of macrophages against Mtb provides insight into the pathogenesis of TB and a host‐directed therapeutic target for future TB treatment.

## 4 Methods and Materials

### 4.1 Bacteria and Culture

Mtb strains H37Ra (ATCC 25177), H37Rv (ATCC 27294), H37Rv‐GFP, and BCG were incubated at 37°C in Middlebrook 7H9 liquid medium supplemented with 10% OADC (oleic acid; Sigma #O1008, albumin; Sigma #A9647, dextrose; Sigma #G7528, and catalase; Sigma #C1354), 5% glycerol (DUKSAN #781), and 0.05% Tween‐80 (SAMCHUN #9005‐65‐6), with gentle agitation. Aliquots with a concentration of 1 × 10^8^ CFU/mL were stored at −80°C until use.

Lm (NCCP14714) was provided by the National Culture Collection for Pathogens, the National Institute of Infectious Diseases, and the National Institute of Health. Lm was incubated at 37°C in brain–heart infusion medium supplemented with erythromycin (5 µg/mL) and streptomycin (50 µg/mL). The optical density of the Lm culture was measured at 600 nm, and an OD_600_ of 1.0 was considered as 2 × 10^9^ CFU/mL.

### 4.2 Mice and In Vivo Transfection and Infection

Six‐week‐old female C57BL/6 mice were purchased from Nara Biotech (Seoul, Republic of Korea) and maintained in a specific pathogen‐free barrier at the Preclinical Experiment Center, College of Medicine, Chungnam National University, Daejeon, Republic of Korea. All experiments were performed using 6‐week‐old female C57BL/6 mice.

For the in vivo transfection experiment, mice were transfected with control siRNA, siG3bp1, or siG3bp2 (Bioneer, #27041‐3, #23881‐2) using Invivofectamine 3.0. Briefly, siRNAs at a concentration of 2.4 mg/mL were mixed with a complexation buffer in a 1:1 ratio. The diluted siRNAs were then combined with Invivofectamine 3.0 reagent in a 1:1 ratio (v/v) and incubated at 50°C for 30 min. The resulting siRNA complexes were 1/6 diluted in phosphate‐buffered saline (PBS). The final siRNA complexes were intravenously injected at a concentration of 1.8 mg/kg. Two days after the transfection, mice were intratracheally infected by H37Rv with 1 × 10^6^ CFU/50 µL PBS or control PBS, following anesthesia with an intraperitoneal (i.p.) injection of avertin (375 mg/mL/kg; Sigma #T48402). Seven days postinfection, the mice were transfected with siRNAs at a concentration of 0.6 mg/kg, and another 7 days later, the mice were sacrificed for further experiments.

ISRIB solution and vehicle were prepared by dissolving in DMSO and PEG400 (1:1 mixture). ISRIB was administered at 0.25 mg/kg by i.p., with a single dose given 2 days before Mtb infection and additional dose administered 7 days after the infection.

### 4.3 BMDM Isolation and In Vitro Infection

Bone marrow cells were isolated from the femurs and tibias of female C57BL/6 mice (6 weeks old) and differentiated into BMDMs by culturing in Dulbecco's modified Eagle's medium (DMEM, Welgene) supplemented with 10% fetal bovine serum (FBS, Welgene), 5% antibiotics, and macrophage colony‐stimulating factor (25 ng/mL; R&D systems #416‐ML) for 4 days. After differentiation, adherent BMDMs were washed and allowed to rest in DMEM containing 5% FBS for 1 day before infection.

For Mtb infection, the cells were infected for 3 h, and nonphagocytosed mycobacteria were washed out, followed by further incubation in DMEM supplemented with 5% FBS. All infections not specifically mentioned in the figure legends were induced with the virulent strain H37Rv at an MOI of 1.

For Lm infection, the cells were infected with Lm at an MOI of 1 for 1 h. Then, the cells were washed with PBS and further incubated in DMEM with 5% FBS containing 50 µg/mL of gentamicin.

### 4.4 LC–MS/MS Sample Preparation

The LC–MS/MS sample preparation was performed by EBIOGEN (Republic of Korea). Briefly, cell lysates were quantified using a BCA assay, and a total of 100 µg of proteins were subjected to filter‐aided sample preparation digestion. Samples were reduced with 5 mM tris(2‐carboxyethyl)phosphine for 30 min at 37°C and alkylation with 50 mM iodoacetamide for 1 h at 25°C in the dark. After centrifugation at 14,000 × *g* for 15 min, 100 µL of 8 M urea (0.1 M Tris/HCl, pH 8.5) was added and centrifuged three times at 14,000 × *g* for 15 min. For digestion, 100 µL of 50 mM ammonium bicarbonate was added and centrifuged three times at 14,000 × *g* for 15 min. Trypsin in 50 mM ammonium bicarbonate was added to the protein sample to a final concentration of 2% (v/v) and incubated for 18 h at 37°C. The digested peptides were vacuum filtered twice with 40 µL of 50 mM ammonium bicarbonate. The digestion was stopped by adding 15 µL of formic acid (pH 2.0) to the resulting filtrate. The digested peptides were desalted using a C_18_ Micro‐Spin‐Column (Thermo Fisher Scientific). The column was prepared by adding 100 µL of MeOH, 100 µL of 0.1% formic acid, 100 µL of 80% acetonitrile, and 100 µL of 0.1% formic acid. The samples were loaded into the column, followed by the addition of 50 µL of 0.1% formic acid. The samples were eluted by 100 µL of 80% acetonitrile, dried, and stored at −20°C.

### 4.5 LC–MS/MS Analysis and Proteomics Analysis

The LC–MS/MS proteomic analysis was performed using a Q‐Exactive mass spectrometer (Thermo Fisher Scientific) at EBIOGEN. Briefly, Peptides were loaded to the column (trapping column C_18_, 3 µm, 100 Å, 75 µm × 2 cm; analytical column PepMap RSLC C_18,_ 2 µm, 100 Å, 75 µm × 50 cm) with buffer A (0.1% formic acid) and buffer B (80% acetonitrile with 0.1% formic acid) at a flow rate of 300 nL/min. The *m*/*z* range was set to 400–2000 *m*/*z*. LC–MS/MS data were analyzed using Proteome Discoverer (Thermo Fisher Scientific) based on a mouse database from Uniprot (proteome ID UP000000589). Functional annotation clustering and GO analysis were performed using the DAVID Bioinformatics Resources (https://david.ncifcrf.gov/home.jsp) with default parameters and plotted using GraphPad Prism 10. STRING analysis was performed using the STRING tool v11.5 (http://string‐db.org).

### 4.6 H&E Staining

Mouse lung tissue was perfused with 3.5% formaldehyde (SAMCHUN #50‐00‐0) for 3 min and embedded in paraffin. The tissues were sliced into 5 µm thickness using a microtome, and the tissue sections were mounted on glass slides. The slides were deparaffinized with xylene (DUKSAN #115) and hydrated with ethanol. Slides were incubated in Gill's Hematoxylin V (Muto Pure Chemicals #20032) solution for 5 min, decolorized with 0.3% HCl for 15 s, and rinsed with Scott's blue solution for 7 min. Slides were stained with Eosin Y (Muto Pure Chemicals #3200‐2) for 2 min, dehydrated, cleared, and mounted.

### 4.7 Antibodies

The following primary antibodies were used for immunoblot analysis: mouse anti‐Eif3η (SCT, sc‐137214), mouse anti‐G3bp1 (SCT, sc‐365338), mouse anti‐Lamp1 (SCT, sc‐20011), mouse anti‐Mfn1 (Invitrogen, MA5‐24789), mouse anti‐mTOR (CST, 4517), mouse anti‐Pabp1 (SCT, sc‐166381), mouse anti‐Raptor (SCT, sc‐81537), mouse anti‐Tomm20 (Sigma, WH0009804M1), rabbit anti‐4E‐BP1 (CST, 9644), rabbit anti‐4E‐BP1‐pT37/46 (CST, 2855), rabbit anti‐β‐Actin (CST, 4970), rabbit anti‐Drp1 (CST, 8570), rabbit anti‐Eif2α (CST, 5324), rabbit anti‐Eif2α‐pS51 (CST, 3597), rabbit anti‐Eif4e (CST, 2067), rabbit anti‐G3bp1 (CST, 61559), rabbit anti‐mTOR‐pS2448 (CST, 5536), rabbit anti‐Ndufa12 (abcam, ab192617), rabbit anti‐Opa1 (CST, 80471), rabbit anti‐p70 S6 Kinase (CST, 2708), rabbit anti‐p70 S6 Kinase‐p389 (CST, 9234), rabbit anti‐SQSTM1/p62 (CST, 23214), rabbit anti‐TSC2 (CST, 4308), rabbit anti‐Astrin (Proteintech, 14726‐1‐AP), rabbit anti‐CoxIV (abcam, ab16056), rabbit anti‐Eif4b (CST, 3592), rabbit anti‐Eif4b‐pS406 (CST, 5399), rabbit anti‐G3bp2 (CST, 31799), rabbit anti‐LC3 (MBL, PM036), rabbit anti‐Parkin (CST, 2132), rabbit anti‐Rack1 (abcam, ab62735), and rabbit anti‐TSC1 (Thermo, PA5‐20131).

The following primary antibodies were used for immunofluorescence analysis: mouse anti‐α‐Tubulin (CST, 3873), rabbit anti‐β‐Actin (CST, 4970), rabbit anti‐G3bp1 (abcam, ab181150), mouse anti‐Eif3η (SCT, sc‐137214), mouse anti‐G3bp1 (SCT, sc‐365338), rabbit anti‐G3bp2 (CST, 31799), rabbit anti‐Rack1 (abcam, ab62735), mouse anti‐Pabp1 (SCT, sc‐166381), mouse anti‐Puromycin (Millipore, MABE343), mouse anti‐Raptor (SCT, sc‐81537), rabbit anti‐mTOR (CST, 2983), rabbit anti‐Astrin (Proteintech, 14726‐1‐AP), and rabbit anti‐Ndufa12 (abcam, ab192617).

The following secondary antibodies were used for immunoblot or immunofluorescence analysis: anti‐Mouse IgG HRP (Millipore, 401215), anti‐Rabbit IgG HRP (CST, 7074), Donkey anti‐Mouse IgG Alexa Fluor 647 (Invitrogen, A31571), Goat anti‐Mouse IgG Alexa Fluor 594 (Invitrogen, A‐11005), Goat anti‐Mouse IgG Alexa Fluor Plus 488 (Invitrogen, A32723), Goat anti‐Rabbit IgG Alexa Fluor Plus 488 (Invitrogen, A32731), Goat anti‐Rabbit IgG Alexa Fluor Plus 647 (Invitrogen, A32733), and Goat anti‐Rat IgG Alexa Fluor Plus 488 (Invitrogen, A48262).

### 4.8 Confocal Microscopy Analysis—Tissues

Mouse lung tissues were perfused with 3.5% formaldehyde for 3 min and embedded in paraffin blocks. The paraffin‐embedded tissues were sliced into 5 µm thickness and mounted on glass slides. The slides were then heated in a 55°C oven to dissolve the paraffin, followed by deparaffinization using xylene. Deparaffinized slides were hydrated using ethanol and washed with running tap water for 2 min. Permeabilization was performed using 0.5% Triton X‐100 for 10 min at RT, followed by washing with PBS for 5 min. For antigen retrieval, the slides were placed in a glass jar containing citrate buffer and boiled for 20 min. The slides were immunostained and incubated with 0.1% Sudan Black B (0.1 g/100 µL in 70% EtOH; Sigma #SR2135‐050‐60) for 30 min. The slides were mounted and analyzed using a confocal microscope (Zeiss LSM900). Representative images are shown in all figures.

### 4.9 Confocal Microscopy Analysis—Cells

The cells were washed three times with PBS and fixed with 4% paraformaldehyde (PFA) for 10 min. Cells were permeabilized with 0.5% Triton X‐100 for 15 min, washed with PBS containing 0.1% Tween‐20 (PBST), and blocked with 1% bovine serum albumin (BSA) for 1 h. After blocking, the antibodies were diluted in 1% BSA and incubated with the cells for 2 h at RT. Secondary antibodies were prepared with 1% BSA and incubated with the cells for 1 h at RT, followed by washing with PBST and mounting. Images were captured using a Zeiss LSM 900 confocal microscope. For Z‐stack analysis and 3D reconstruction, Z‐stack images (0.15 µm step size) were captured using a Zeiss LSM 900 confocal microscope and analyzed using ZEN Microscopy Software v3.2. Dual‐color pixel analysis was performed with the default value of colocalization analysis in ZEN software, and Pearson correlation coefficient values were obtained.

For SYTO9 staining of Lm, Lm‐infected BMDMs were fixed, permeabilized, blocked, and incubated with the above‐mentioned antibodies. During secondary antibody incubation, 50 nM SYTO9 was added to the solution.

### 4.10 Immunoblot Analysis

The cells were washed with PBS and lysed using radioimmunoprecipitation assay buffer supplemented with a Protease Inhibitor Cocktail (PIC; Roche #11697498001). For the in vivo samples, lung tissues were homogenized and lysed in radioimmunoprecipitation assay buffer containing PIC. For both the in vitro and in vivo samples, the protein concentration was measured using Bradford Dye Reagent (BIORAD #5000205)t. Protein samples were prepared by mixing lysates with 5 × sample buffer, followed by heating for 10 min at 95°C. The protein samples were subjected to sodium dodecyl sulfate‐polyacrylamide gel electrophoresis. Subsequently, the separated proteins were transferred onto polyvinylidene difluoride membranes, which were then blocked with 3% skim milk. The membranes were incubated with primary antibodies overnight at 4°C. The following day, the membranes were washed with TBST and incubated with horseradish peroxidase‐conjugated secondary antibodies. A chemiluminescent horseradish peroxidase substrate was used and detected using a ChemiDoc XRS+. The captured images were analyzed using Image Lab v6.0.1.34.

### 4.11 RNA Fluorescence In Situ Hybridization

RNA fluorescence in situ hybridization was performed according to the manufacturer's instructions. Briefly, the cells were fixed with Fixation Buffer and permeabilized with 70% EtOH. The cells were then washed with Wash Buffer A and incubated with Hybridization Buffer containing the T30‐ATTO 647N probe and GFP‐G3bp1 antibody for 4 h. After incubation, the cells were washed with Wash Buffer A and then stained with DAPI, followed by washing with Wash Buffer B and mounting.

### 4.12 In Vitro siRNA Transfection and Treatment

After 4 days of differentiation into BMDMs, the cells were transfected with siRNAs using Lipofectamine 3000 (Invitrogen #3000001) according to the manufacturer's instructions. Prior to transfection, the medium was replaced with plain DMEM, and an siRNA (200 nM)–lipofectamine complex was prepared in Opti‐MEM. The siRNA mixture was incubated at RT for 15 min and then added dropwise to the BMDMs. After 4 h, the medium was replaced with DMEM containing 5% FBS, and the cells were incubated for 48 h before Mtb infection.

To inhibit phagocytosis, the cells were treated with 2 µM Cyto D at 1 h before infection. For the ISRIB (Sigma #SML0843) treatment, BMDMs were grown in 5% FBS DMEM and treated with 1.5 µM ISRIB for 1 h prior to Mtb infection. To manipulate mTOR activity, BMDMs were treated with the following chemicals at 0 hpi: MHY1485 (MHY; Sigma #SML0810) at 15 µM, and rapamycin (Rapa; Sigma #R8781) at 400 nM. The ROS scavengers: NAC 0.5 mM (Sigma #A7250), 2‐mercaptoethanol 100 µM, and MitoTEMPO 100 µM (Sigma #SML0737), were treated 1 h prior to Mtb infection. To inhibit glycolysis, BMDMs were washed with PBS and maintained in a glucose‐free medium supplemented with glucose and 2‐DG (Sigma #D8375) at the indicated concentrations for 1 h.

Empty liposomes and ATPsomes (Encapsula NanoScience #APS‐208) were used according to the manufacturer's instructions. Liposomes comprising a 3:7 ratio of phosphatidylserine to phosphatidylcholine were dissolved in DW before use. The resulting liposomes were approximately 100 nm in diameter and contained 1 mM ATP. ATP‐containing ATPsomes (10 mM) and an equivalent volume of empty liposomes were added to the media at the indicated times.

### 4.13 ATP Measurement

Intracellular ATP concentration was measured using an ATP assay kit (Abcam). Briefly, the cells were washed with cold PBS, harvested, and lysed with ATP assay buffer. The lysate was gently resuspended and centrifuged at 13,000 rpm for 5 min. The supernatant was added to a 96‐well plate in triplicate. The reaction mixture was prepared as follows: ATP assay buffer 44 µL, ATP probe 2 µL, ATP converter 2 µL, and developer mix 2 µL (total of 50 µL). The reaction mixture was added to the well containing the cell lysate, followed by incubation for 30 min under protection from light. Fluorescence was measured at excitation/emission wavelengths of 535/587 nm.

### 4.14 Intracellular Survival Assay

To collect the intracellular mycobacteria, the cells were washed with PBS and lysed using autoclaved DW. Lysates were serially diluted with autoclaved DW and plated in triplicate on Middlebrook 7H10 agar plates. The plates were incubated at 37°C for 14 days, and the resulting colonies were counted. At least three independent experiments were performed, and the mean ± SEM was plotted.

For Lm, cells were washed, lysed, diluted, and plated on brain–heart infusion agar plates. The plates were incubated at 37°C for 1 day, and the resulting colonies were counted. At least three independent experiments were performed, and the mean ± SEM was plotted.

For the intracellular survival assay in mouse lungs, lung tissues were isolated and washed with PBS. The tissues were homogenized with autoclaved DW and filtered with a 70 µm cell strainer. The lysates were serially diluted and plated in triplicate on Middlebrook 7H10 agar plates. The plates were incubated for 2 weeks, and the colonies were counted. Six siControl‐transfected mice and seven siG3bp1/2‐transfected mice were analyzed, and the graph was plotted as mean ± SEM.

### 4.15 Ribopuromycylation Assay

A ribopuromycylation assay was conducted to measure de novo protein synthesis. Prior to lysis, cells were treated with puromycin (5 µg/mL) for 10 min at 37°C to label the nascent polypeptide chains. Immunoblotting was performed on the cell lysates, and de novo protein synthesis was detected using an antipuromycin antibody. The band intensities of puromycin were quantified by performing at least three independent experiments using Image Lab v6.0.1.34.

For confocal microscopy analysis of the ribopuromycylation assay, the cells were treated with puromycin (5 µg/mL) before fixation with 4% PFA for 10 min. The cells were permeabilized and blocked with 1% BSA, followed by immunofluorescence staining with an antipuromycin antibody. Confocal microscopy was used to detect puromycin signals, and the fluorescence signal was pseudoscaled to a rainbow color scheme to visualize and quantify the signal intensity. The fluorescence intensities of the segmented cells from three independent experiments were quantified using ZEN Microscopy Software v3.2.

### 4.16 Coimmunoprecipitation Analysis

Whole‐cell lysates were prepared in lysis buffer (50 mM Tris–Cl [pH 8.0], 150 mM NaCl, 1% Triton X‐100, 1.5 mM EDTA [pH 8.0], and PIC). Cell lysates containing 0.8 mg of protein were incubated overnight with anti‐G3bp1, TSC1, TSC2, and anti‐control IgG. The next day, Dynabead Protein A was added and incubated for 2 h with rotation. The protein–bead complexes were washed three times with lysis buffer. The complexes were then resuspended in 1 × sample buffer and heated at 95°C for 10 min. The 1% input and immunoprecipitation samples were subjected to immunoblotting.

### 4.17 Proximity Ligation Assay (PLA)

The proximity ligation assay (PLA) was performed according to the manufacturer's instructions (Sigma #DUO92008). The cells were washed with PBS, fixed with 4% PFA, and washed three times with PBST. Cells were blocked with blocking solution for 30 min at 37°C. After blocking, cells were washed with PBST and incubated with primary antibodies in antibody diluent for 2 h at 37°C. Subsequently, cells were washed with Wash Buffer A two times, and 5 × PLA Probes Anti‐Rabbit PLUS and Anti‐Mouse MINUS were diluted with antibody diluent and incubated with the cells for 1 h at 37°C. The ligation solution prepared with ligase and 1 × ligation buffer was added to the cells after incubation with the probes. Cells were incubated with the ligation solution for 30 min at 37°C, followed by a wash with Wash Buffer A. Polymerase was added in 1 × amplification buffer and incubated with cells for 100 min at 37°C. The cells were washed with Wash Buffer B, stained with DAPI to visualize the nuclei, and mounted. PLA puncta were quantified using ImageJ/FIJI software and manually validated. At least 100 cells were analyzed in all PLA experiments. Representative images are shown in all figures.

### 4.18 ROS Measurement

The BMDMs were treated with trypsin for 5 min and resuspended in PBS. The cells were then centrifuged at 7000 rpm for 30 s. Pellets were washed with PBS and treated with 20 µM dihydroethidium (DHE; Sigma #D1168) for 30 min at 37°C. After three washes with PBS, the cells were fixed in 4% PFA. ROS levels were measured using a NovoCyte 3000 flow cytometer and analyzed using FlowJo Software v10.8. All experiments were performed in triplicate.

### 4.19 Griess Assay

NO formation was investigated by measuring nitrite (NO_2_^−^), which is the primary stable breakdown product of NO, following the manufacturer's instructions for the Griess Reagent System (Promega #G2930). BMDM culture media were filtered and added to a 96‐well plate in triplicate. A standard solution was prepared by serial dilution from 100 to 0 µM. The sulfanilamide solution was added to the standard and culture media and incubated for 10 min under protection from light. After 10 min, NED buffer was added, and the cells were incubated for 10 min at RT while protected from light. Absorbance was measured using a microplate reader with a 520 nm filter. Three independent experiments were performed, and the results were analyzed.

### 4.20 LDH Assay

The cell viability was assessed using a lactate dehydrogenase (LDH) assay (Sigma MAK529) following the manufacturer's protocol. Briefly, the culture media from Mtb‐infected or other experimental condition‐treated macrophages was collected, and LDH release was measured. The collected supernatant was incubated with the reaction mixture for 30 min. The absorbance was measured at 490 nm. Data were normalized to the values of the control groups. All experiments were conducted in triplicate.

### 4.21 Cytometric Bead Array

Cytokine production was quantified in the culture media of BMDMs or mouse serum using a cytometric bead array, following the manufacturer's instructions (BD #552364). In brief, a 50 µL standard solution was prepared by serial dilution from 5000 to 0 pg using an assay diluent, and 50 µL of the filtered culture media of BMDMs or serum was prepared in triplicate. Mouse MCP‐1 and TNF capture beads were added to the standard and sample tubes, followed by the addition of 20 µL of PE detection reagent. The mixture was incubated in the dark at RT for 2 h. The mixture was washed with wash buffer and centrifuged at 1500 rpm for 5 min. The resulting beads were resuspended in wash buffer and transferred to round‐bottomed tubes for FACS analysis.

For the FACS analysis, the setup beads were first analyzed to determine the mean fluorescence range of each bead. Intact beads were gated as P1 using an FSC versus SSC dot plot. The PE versus APC dot plot separated the P2 and P3 populations, whereas the FITC versus APC dot plot separated the P4 population. The mean fluorescence intensity (MFI) for each population was adjusted as follows: P2: APC 70000; P3, PE 75; and P4, FITC 75. After adjusting the MFI range using the setup beads, the standard and samples were analyzed by measuring the fluorescence signal from 300 beads for each cytokine. The results were further processed using FCAP Array software. A mouse inflammatory cytokine bead library was established, and the resulting files were assigned to bead clusters, filtered to remove debris, and quantified.

### 4.22 Mitochondria Analysis

For the mitochondrial function assay, BMDMs were seeded onto Seahorse XF96 cell culture microplates (Agilent Technologies) at a density of 5 × 10^4^ cells per well. After Mtb infection for the indicated periods, the infected cells were washed twice with XF Base Medium supplemented with 25 mM glucose and 1 mM sodium pyruvate. Mitochondrial oxygen consumption rates were measured using the Seahorse XF96 and analyzed with the following drug injections: port A, oligomycin 1.5 µM; port B, FCCP 1.0 µM; and port C, Rot/AA 0.5 µM. Mitochondrial parameters were calculated according to the manufacturer's instructions.

A mitochondrial membrane potential assay was performed using JC‐1 staining. After infection, cells were treated with 1 µM JC‐1 and incubated at 37°C for 30 min. Red and green fluorescence were measured at excitation/emission wavelengths of 550/600 nm for red and 485/535 nm for green using a fluorescence plate reader. Red/green fluorescence ratios were calculated and plotted.

Mitochondria were isolated using a Mitochondria Isolation Kit for Cultured Cells (Thermo Fisher Scientific). Briefly, cell pellets were lysed with Mitochondria Isolation Reagent A by vortexing at medium speed, followed by incubation on ice. Then, 10 µL of Mitochondria Isolation Reagent B was added, followed by the addition of 800 µL of Mitochondrial Isolation Reagent C and inverting. After centrifugation, the supernatant was transferred to a new tube and centrifuged at 3000 × *g* for 15 min. The resulting supernatant contained the cytosolic fraction, and the pellet contained isolated mitochondria. The fractions were further analyzed by immunoblotting and mitochondrial complex I activity assays.

Mitochondrial complex I activity was determined using a Complex I Enzyme Activity Microplate Assay Kit (Abcam). Isolated mitochondria were subjected to sample preparation, and mitochondrial protein (1.25 µg/µL) samples were prepared with detergent solution. Then, the samples (200 µL) were loaded onto 96‐well plates precoated with capture antibodies specific for Complex I, followed by incubation for 3 h at RT. The plate was washed twice with the Wash Buffer. The Assay Solution was prepared as follows: Dilution Buffer (3.29 mL), 20 × NADH (175 µL), and 100 × Dye (35 µL). The Assay Solution was added to the plate, and the kinetics were measured with the following parameters: wavelength 450 nm, time 60 min, interval 30 s, and shaking between readings at RT. Additionally, 1 µM of rotenone was treated for 1 h as the positive control.

### 4.23 Monitoring Autophagic Flux

Autophagic flux was measured using a tandem fluorescence LC3 plasmid (tfLC3). The cells were transfected with tfLC3 for 48 h, followed by infection with Mtb for 24 h or incubation in LG medium with or without 200 nM bafilomycin A1. Subsequently, the cells were fixed with 4% PFA, mounted, and examined using confocal microscopy. Finally, quantification involved counting the number of yellow puncta (autophagosomes) and red puncta (autolysosomes).

### 4.24 Immunofluorescence Quantification

To quantify the MFI, images were captured using a Zeiss LSM 900 confocal microscope. The images were further analyzed using ZEN software with default settings of arithmetic mean intensity for each channel and exported to Microsoft Excel. At least three independent experiments were performed. All fields of view were selected and quantified in a blinded manner. The resulting values were plotted on graphs as mean ± SD.

To analyze the Mtb area in the cells, images of Mtb‐infected BMDMs were acquired in triplicate using a Zeiss LSM 900 confocal microscope with five fields of view per sample. The cells were segmented based on DAPI using ZEN software. The Mtb area in each cell type was determined using the GFP channel. At least 100 cells were analyzed and plotted on a violin plot with medians and quartiles.

### 4.25 SG Quantification

To quantify the ratio of SG‐positive cells and the number of SGs, the acquired images were analyzed on a percentage cell basis, as described in a previous study [78], using the ImageJ/FIJI software, followed by manual validation. At least 15 fields of view were analyzed in triplicate, and the results were plotted as mean ± SD. Unless otherwise specified in the figure legend, SG quantification is based on the immunostaining of G3bp1.

### 4.26 Statistical Analysis

All statistical analyses were performed as follows: A two‐component hypothesis test was performed using the unpaired *t*‐test. Multiple variable testing was performed using one‐way analysis of variance (ANOVA), followed by Tukey's post hoc test. *p* values of < 0.05*, 0.01**, 0.001***, and 0.0001**** were considered significant. All experiments were independently repeated at least three times. All data are presented as mean ± SD, unless otherwise indicated, in the figure legends. For correlation analysis (Figure 3D), a normal distribution was determined using the Shapiro–Wilk test, and Spearman correlation coefficients (*ρ*) were calculated. All data points, including potential outliers, were included in the analysis without exclusion. All statistical analyses were performed using GraphPad Prism version 10.

## Author Contributions

Conceptualization: Jaewhan Kim, Kee K. Kim and Chang‐Hwa Song. Methodology: Jaewhan Kim. Validation: Jaewhan Kim. Formal Analysis: Jaewhan Kim. Investigation: Jaewhan Kim. Resource: Jaewhan Kim, Sang‐Hun Son, Ji‐Ae Choi, Junghwan Lee, Seoyeon Jo, Soo‐Na Cho, Doan Tam Nguyen and Doyi Son. Writing – original draft: Jaewhan Kim. Writing – review & editing: Jaewhan Kim and Chang‐Hwa Song. Visualization: Jaewhan Kim. Supervision: Jaewhan Kim and Chang‐Hwa Song. Project Administration: Chang‐Hwa Song. Funding Acquisition: Chang‐Hwa Song. All the authors have read and approved the final manuscript.

## Funding

This work was supported by grants from National Research Foundation of Korea (NRF) funded by the Korean government (MSIT) (RS‐2025‐00523465 and RS‐2025‐02263643) and by BK21 FOUR Program by Chungnam National University Research Grant, 2024.

## Ethics Statement

All animal procedures were approved by the Institutional Animal Care and Use Committee (IACUC) of Chungnam National University, Republic of Korea (permit number: CNUH‐2023‐IA0028‐00). All animal experiments and protocols were performed in accordance with Korean Food and Drug Administration guidelines.

## Conflicts of Interest

The authors declare no conflicts of interest.

## Supporting information




**Figure S1** GO analysis of differentially expressed proteins in macrophages infected with Mtb. (A) Quantitative proteomics analysis for profiling protein expression. BMDMs were either uninfected (UN) or infected with Mtb for the indicated hours. Whole‐cell lysates were processed for quantitative LC–MS/MS analysis. (B) A heatmap displaying significantly differentially expressed proteins. The average z‐scores of 756 proteins were hierarchically clustered. Fold change (FC) ± 2.0; −log10 (*p* value) > 2. (C–E) Volcano plots illustrate −log10 (*p* value) versus log2 FC of Mtb 12 hpi/UN (C), 24 hpi/UN (D), and 24 hpi/12 hpi (E). (F–I) GO analysis of 356 increased (F) and 191 decreased (G) proteins at 12 hpi compared to UN, and 377 increased (H) and 174 decreased (I) proteins at 24 hpi compared to UN. Proteins with log2 FC ± 1.0 and −log10 (*p* value) > 2 were analyzed. The top five GO terms are shown.
**Figure S2** Proteomic analysis of Mtb‐infected BMDMs highlights SG‐related proteins. (A) A Venn diagram of differentially expressed proteins in BMDMs infected with Mtb for 12 or 24 h from LC–MS/MS analysis. Up‐, down‐, and contraregulated proteins are shown in red, blue, and black. UN: uninfected. (B) Functional annotation clustering of 32 proteins consistently upregulated during Mtb infection from (A). Functional enrichment was conducted using DAVID Bioinformatics Resources, and the top three clusters are shown based on enrichment scores. (C) A heatmap of 18 SG‐assembly proteins among 756 identified proteins. Data were annotated using the MGI database v6.22, and z‐scores are represented by a color gradient.
**Figure S3** Downregulation of autophagosome–lysosome fusion in Mtb‐infected macrophages. (A) GO analysis of proteins that showed decreased abundance in Mtb‐infected BMDMs (related to Figure S1, C and D). The top five are shown. (B) Immunoblot analysis of autophagy markers in Mtb‐infected BMDMs. LC3A/B‐II and p62 accumulate as infection time increases. (C) Immunoblot analysis of autophagy substrate in low‐glucose (LG)‐starved BMDMs treated with MHY1485 or bafilomycin A1 (Baf. A1). (D) Immunoblot analysis of autophagy substrate in Mtb‐infected WT and SGneg BMDMs or MHY1485‐treated BMDMs. (E) Confocal microscopy images of tfLC3‐transfected BMDMs. Cells were either infected with Mtb or starved in LG for 24 h. The arrowhead indicates the autolysosome. Scale bar indicates 10 µm. (F) Quantification of yellow puncta and red puncta from (E). The bar indicates the total number of yellow and red puncta in a cell. Each color represents a portion of yellow and red puncta. A total of 20 cells were analyzed. Data are presented as mean ± SD. n.s., nonsignificant, **p < 0.01, compared with LG, using Kruskal–Wallis test followed by Dunn's post hoc test. (G) Immunofluorescence analysis of LAMP1 (green) and LC3 (magenta) in RFP‐Mtb‐infected BMDMs treated with MHY1485 or rapamycin (Rapa.) for 24 h. The red dotted image indicates RFP‐positive infected cells, while the white dotted image indicates RFP‐negative uninfected cells. Nuclei: blue. Scale bar indicates 10 µm. Representative images are shown.
**Figure S4** SG deficiency does not activate compensatory stress responses during Mtb infection. (A–C) Relative mRNA expression levels of genes involved in the ISR/Eif2α axis (A), Nrf2‐mediated oxidative stress axis (B), and UPR axis (C) were measured in uninfected (UN) or Mtb‐infected BMDMs transfected with siControl or siG3bp1, 2. Data are normalized to β‐Actin and presented as mean ± SD (*n* = 3). n.s., nonsignificant, *p < 0.05, **p < 0.01; *t*‐test.
**Figure S5** Double knockdown of G3bp1 and G3bp2 did not alter cap‐dependent mRNA translation and immune responses of macrophages. (A) Immunofluorescence analysis of the uninfected G3bp1/2 dKD BMDMs. G3bp1 (green) and G3bp2 (red). Nuclei: blue. Scale bar indicates 5 µm. (B) Immunofluorescence analysis of ribopuromycylation in uninfected G3bp1/2 dKD BMDMs. Puromycin (white). Nuclei: blue. Scale bar indicates 10 µm. (C) Quantification of puromycin signal intensity from (B). (D) Immunoblot analysis of the uninfected G3bp1/2 dKD BMDMs. (E) Total ROS from uninfected BMDMs were measured by DHE staining. (F) Nitrite production was measured in uninfected BMDMs culture media. (G) Quantification of TNF and MCP‐1 production in the uninfected BMDMs culture media.
**Figure S6** SGs form independently of virulence and ROS, with minor antigen contribution. (A) Immunofluorescence analysis of SGs in BMDMs infected by BCG, H37Ra, or H37Rv with a MOI of 1 for 24 h and treated with H37Rv culture filtrate proteins (CFPs), or virulent antigens of H37Rv for 24 h. Nuclei: blue; G3bp1: green; α‐Tubulin: red. Scale bar indicates 10 µm. (B) Quantification of number of SGs per cell from (A). n.s., nonsignificant, *p < 0.05, **p < 0.01, ***p < 0.001, ****p < 0.0001; compared with UN in black label; compared with H37Rv only in red label; One‐way ANOVA followed by Tukey's multiple comparison post hoc test. (C) Quantification of SG+ cell ratio from (A). n.s., nonsignificant, *p < 0.05, **p < 0.01, ***p < 0.001, ****p < 0.0001; compared with UN in black label; compared with H37Rv only in red label; One‐way ANOVA followed by Tukey's multiple comparison post hoc test. (D) Immunofluorescence analysis of phagosomal damage using GAL‐3 staining in siRNA‐transfected BMDMs infected by RFP‐H37Rv with a MOI of 1 for 24 h. Scale bar indicates 5 µm. (E) Quantification of number of GAL‐3 puncta per cell from (D). n.s., nonsignificant; Mann–Whitney test. (F) Immunofluorescence analysis of SGs in BMDMs infected by H37Rv with a MOI of 1, 3, and 5 for 24 h. Nuclei: blue; G3bp1: green. Scale bar indicates 20 µm. (G) Quantification of SG+ cell ratio from (F). n.s., nonsignificant. (H) Immunofluorescence analysis of SGs in H37Rv‐infected BMDMs with ROS scavengers: NAC 0.5 mM, 2‐mercaptoethanol (BME) 100 µM, and MitoTEMPO 100 µM. Nuclei: blue; G3bp1: green. Scale bar indicates 10 µm. (I) Quantification of number of SGs per cell from (H). n.s., nonsignificant, ****p < 0.0001; compared with UN in black label; compared with H37Rv only in red label; One‐way ANOVA followed by Tukey's multiple comparison post hoc test. (J) Quantification of SG+ cell ratio from (H). n.s., nonsignificant, ****p < 0.0001; compared with UN in black label; compared with H37Rv only in red label; One‐way ANOVA followed by Tukey's multiple comparison post hoc test.
**Figure S7** Inverse correlation between cellular ATP levels and SG dynamics. (A) Immunofluorescence analysis of uninfected BMDMs treated with Rot/AA (0.1 µM, LOW; 1.0 µM, MID; 2.0 µM, HIGH) or ATPsome (2.5 mM, LOW; 5.0 mM, MID; 10 mM, HIGH) for 24 h. Cells were stained for G3bp1 (green) and nuclei (blue). Scale bar 20 µm. (B) Immunofluorescence analysis of Mtb‐infected BMDMs treated with Rot/AA (0.1 µM, LOW; 1.0 µM, MID; 2.0 µM, HIGH) or ATPsome (2.5 mM, LOW; 5.0 mM, MID; 10 mM, HIGH) for 24 h. Cells were stained for G3bp1 (green) and nuclei (blue). Scale bar 20 µm. (C) Quantification of intracellular ATP concentration (gray) and SG number per cell (green) under different conditions from (A and B). Data represent mean ± SD (*n* = 15 for ATP; *n* = 50 for SG). (D) Pearson correlation between ATP levels and SG number per cell for uninfected and Mtb‐infected conditions from (A) to (C).
**Figure S8** Lm infection induces ATP reduction and SG formation, thus inhibiting mTORC1 and innate immune activity. (A) Immunofluorescence analysis of the Lm‐infected BMDMs. Cells were infected with Lm (MOI 1) and stained with SYTO9 (Lm) and G3bp1. Scale bar indicates 5 µm. (B) Immunofluorescence analysis of the Lm‐infected BMDMs with or without Cyto D. Cells were infected with Lm (MOI 1) and stained with SYTO9 (Lm) and G3bp1. Scale bar indicates 5 µm. (C) Intracellular ATP concentration of Lm‐infected WT and SGneg BMDMs and Cyto D‐treated BMDMs. n.s., nonsignificant, *p < 0.05, ****p < 0.0001; compared with siCont; One‐way ANOVA followed by Tukey's multiple comparison post hoc test. (D) Immunoblot analysis of Lm‐infected BMDMs undergoing the indicated time of infection. (E) Immunoblot analysis of Lminfected WT and SGneg BMDMs. (F) Total amount of ROS was measured by dihydroethidium staining. WT and SGneg BMDMs were infected with Lm (MOI 1). **p < 0.01, unpaired *t*‐test. (G) Nitrite production was measured in the Lm‐infected WT and SGneg BMDM culture media. *p < 0.05, unpaired *t*‐test. (H and I) Quantification of TNF (H) and MCP‐1 (I) production in the Lm‐infected WT and SGneg BMDM culture media. Data are presented as mean ± SD (*n* = 3). n.s., nonsignificant, ****p < 0.0001; Mann–Whitney test. (J) CFU of Lm in WT and SGneg BMDMs. Data from three independent experiments are shown as mean ± SD. ***p < 0.001, ****p < 0.0001, compared within the same time point; Mann–Whitney test.

**Table S1** Proteomics for total proteomes of UN or Mtb‐infected BMDMs.

**Table S2** Proteomics for total proteomes of WT or SG^neg^ BMDMs (Mtb infected).

**Table S3** Proteome, which is increased in SG^neg^ BMDMs and included in SG proteome.
Sheet 1: Proteome, which is increased in SG^neg^ BMDMs and included in SG proteome.
Sheet 2: Published SG proteome, integrated

**Supporting Video S1** mco270479‐sup‐0005‐VideoS1.mp4

**Supporting Video S2**: mco270479‐sup‐0006‐VideoS2.mp4

**Supporting Video S3**: mco270479‐sup‐0007‐VideoS3.mp4

## Data Availability

All data are included in the manuscript or supplements and are available from the corresponding authors upon reasonable request.
