# Supplementary material for: Host Translational Control by Stress Granules Promotes Mycobacterium tuberculosis Pathogenesis
Source: MedComm (2020). 2025 Nov 10;6(11):e70479. doi: 10.1002/mco2.70479 (PMC12598499; doi:10.1002/mco2.70479)
Supplement: Supplementary file 1 — Figure S1 GO analysis of differentially expressed proteins in macrophages infected with Mtb. (A) Quantitative proteomics analysis for profiling protein expression. BMDMs were either uninfected (UN) or infected with Mtb for the indicated hours. Whole‐cell lysates were processed for quantitative LC–MS/MS analysis. (B) A heatmap displaying significantly differentially expressed proteins. The average z‐scores of 756 proteins were hierarchically clustered. Fold change (FC) ± 2.0; −log10 (p value) > 2. (C–E) Volcano plots illustrate −log10 (p value) versus log2 FC of Mtb 12 hpi/UN (C), 24 hpi/UN (D), and 24 hpi/12 hpi (E). (F–I) GO analysis of 356 increased (F) and 191 decreased (G) proteins at 12 hpi compared to UN, and 377 increased (H) and 174 decreased (I) proteins at 24 hpi compared to UN. Proteins with log2 FC ± 1.0 and −log10 (p value) > 2 were analyzed. The top five GO terms are shown. Figure S2 Proteomic analysis of Mtb‐infected BMDMs highlights SG‐related proteins. (A) A Venn diagram of differentially expressed proteins in BMDMs infected with Mtb for 12 or 24 h from LC–MS/MS analysis. Up‐, down‐, and contraregulated proteins are shown in red, blue, and black. UN: uninfected. (B) Functional annotation clustering of 32 proteins consistently upregulated during Mtb infection from (A). Functional enrichment was conducted using DAVID Bioinformatics Resources, and the top three clusters are shown based on enrichment scores. (C) A heatmap of 18 SG‐assembly proteins among 756 identified proteins. Data were annotated using the MGI database v6.22, and z‐scores are represented by a color gradient. Figure S3 Downregulation of autophagosome–lysosome fusion in Mtb‐infected macrophages. (A) GO analysis of proteins that showed decreased abundance in Mtb‐infected BMDMs (related to Figure S1, C and D). The top five are shown. (B) Immunoblot analysis of autophagy markers in Mtb‐infected BMDMs. LC3A/B‐II and p62 accumulate as infection time increases. (C) Immunoblot analysis of auto [file MCO2-6-e70479-s006.pdf]

Host translational control by stress granules promotes *Mycobacterium tuberculosis* pathogenesis

Running title: SGs regulate macrophage immunometabolism in TB

Jaewhan Kim[1], Sang-Hun Son[1], Ji-Ae Choi[1], Junghwan Lee[1], Seoyeon Jo[1], Soo-Na Cho[1], Doan Tam Nguyen[1], Doyi Son[1], Kee K. Kim[2], and Chang-Hwa Song[1,3,4*]

[1]Department of Medical Science, College of Medicine, Chungnam National University, Daejeon, 35015, Republic of Korea

[2]Department of Biochemistry, College of Natural Sciences, Chungnam National University, Daejeon, 34134, Republic of Korea

[3]Department of Microbiology, College of Medicine, Chungnam National University, Daejeon, 35015, Republic of Korea

[4]Lead contact

[*]Correspondence: songch@cnu.ac.kr

Figure S1. GO analysis of differentially expressed proteins in macrophages infected with Mtb

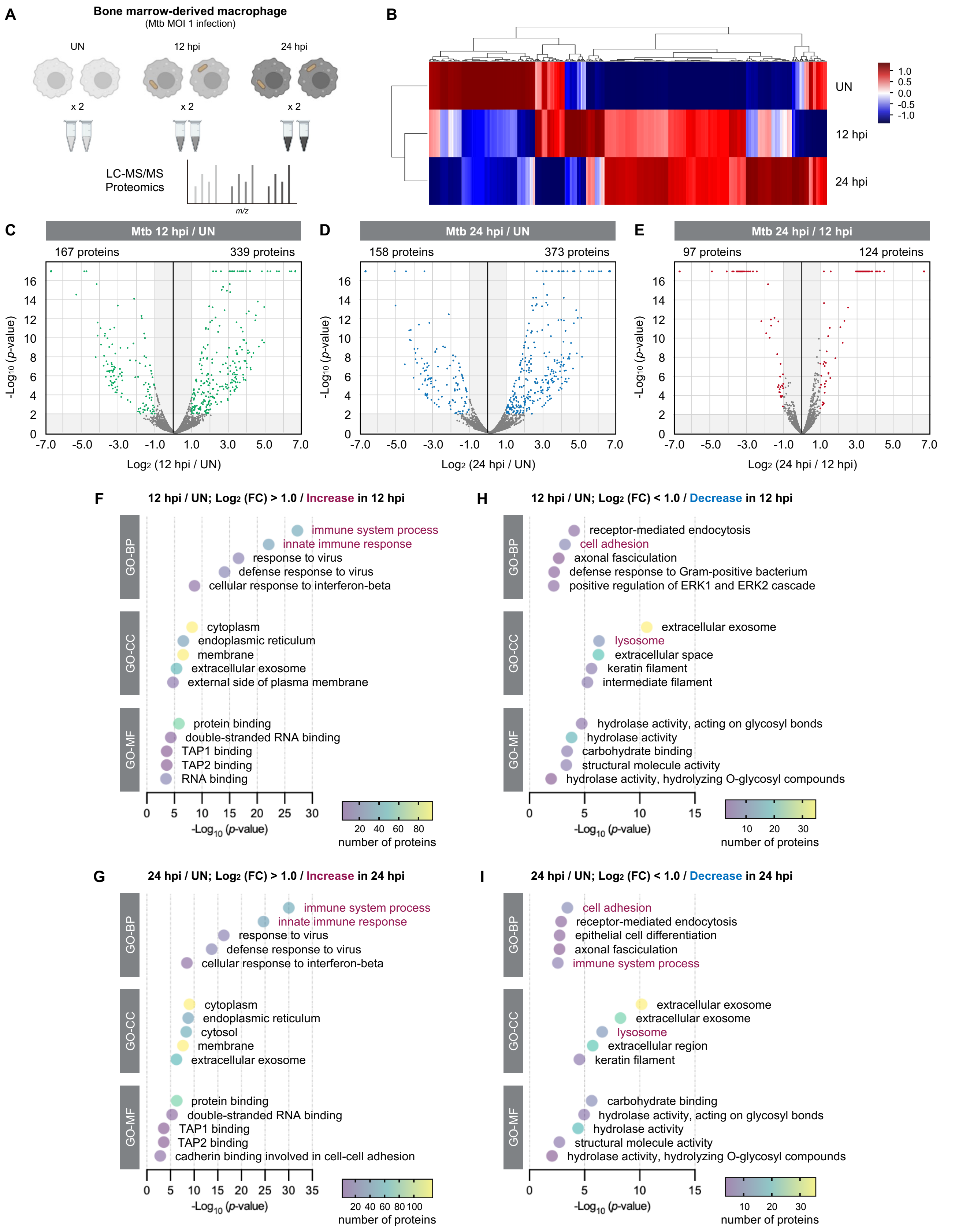

Figure S1. GO analysis of differentially expressed proteins in macrophages infected with Mtb
(A) Quantitative proteomics analysis for profiling protein expression. BMDMs were either uninfected (UN) or infected with Mtb for the indicated hours. Whole-cell lysates were processed for quantitative LC-MS/MS analysis. **(B)** A heatmap displaying significantly differentially expressed proteins. The average z-scores of 756 proteins were hierarchically clustered. Fold change (FC) ± 2.0; -log10 (*p*-value)>2. **(C–E)** Volcano plots illustrate -log10 (*p*-value) versus log2 FC of Mtb 12 hpi/UN (C), 24 hpi/UN (D), and 24 hpi/12 hpi (E). **(F–I)** GO analysis of 356 increased (F) and 191 decreased (G) proteins at 12 hpi compared to UN, and 377 increased (H) and 174 decreased (I) proteins at 24 hpi compared to UN. Proteins with $\log_2$ FC ± 1.0 and -$\log_{10}$ (*p*-value) > 2 were analyzed. The top five GO terms are shown.

Figure S2. Proteomic analysis of Mtb-infected BMDMs highlights SG-related proteins

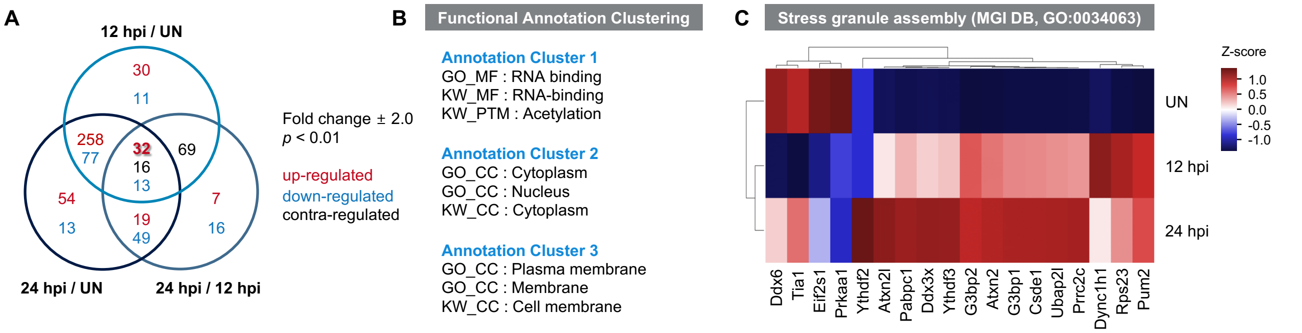

Figure S2. Proteomic analysis of Mtb-infected BMDMs highlights SG-related proteins
(A) A Venn diagram of differentially expressed proteins in BMDMs infected with Mtb for 12 or 24 h from LC-MS/MS analysis. Up-, down-, and contra-regulated proteins are shown in red, blue, and black. UN: uninfected. **(B)** Functional annotation clustering of 32 proteins consistently upregulated during Mtb infection from (A). Functional enrichment was conducted using DAVID Bioinformatics Resources, and the top three clusters are shown based on enrichment scores. **(C)** A heatmap of 18 SG-assembly proteins among 756 identified proteins. Data were annotated using the MGI database v6.22, and z-scores are represented by a color gradient.

Figure S3. Downregulation of autophagosome-lysosome fusion in Mtb-infected macrophages

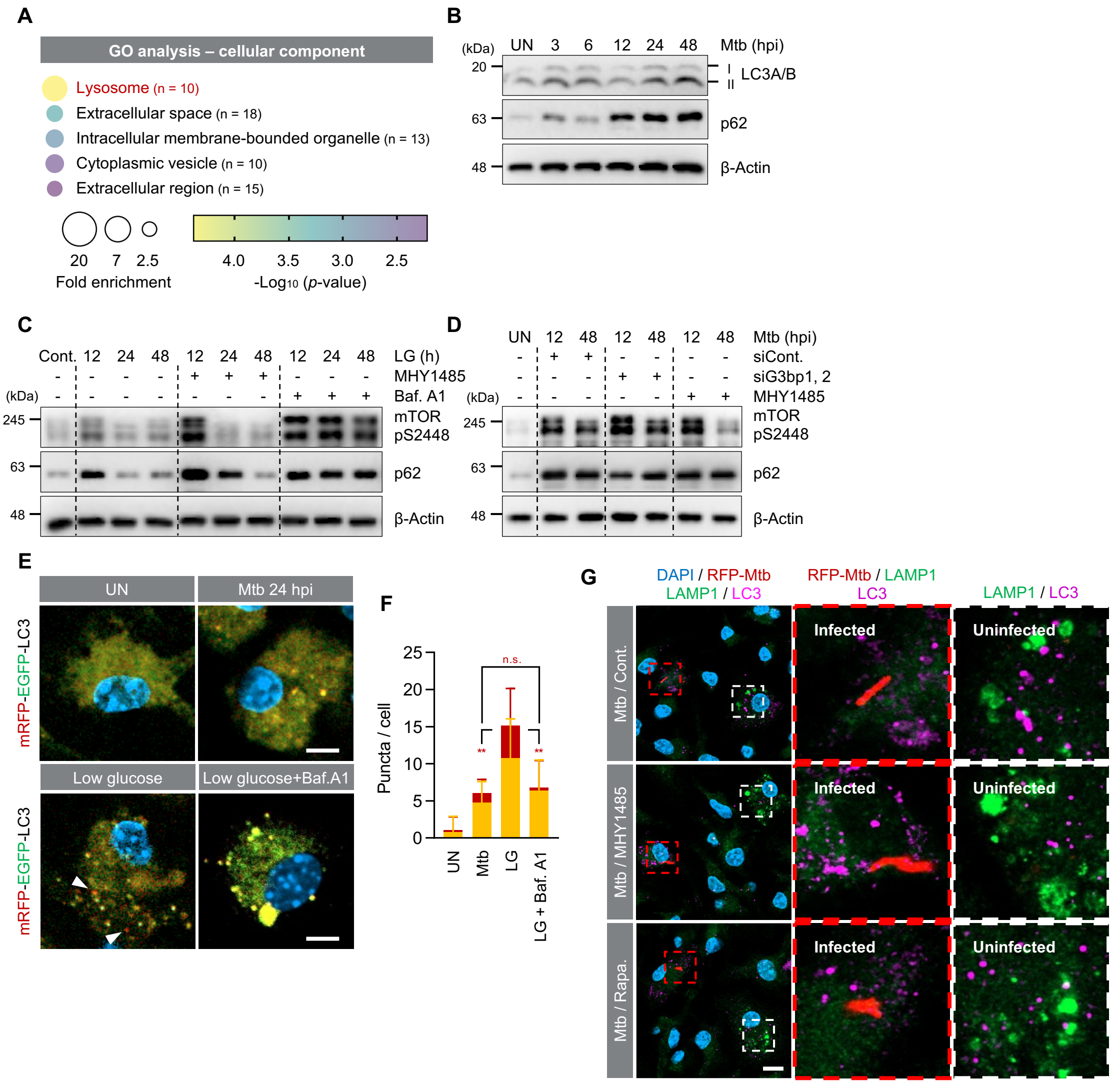

Figure S3. Downregulation of autophagosome-lysosome fusion in Mtb-infected macrophages
(A) GO analysis of proteins that showed decreased abundance in Mtb-infected BMDMs (related to Fig. S1, C and D). The top five are shown. **(B)** Immunoblot analysis of autophagy markers in Mtb-infected BMDMs. LC3A/B-II and p62 accumulate as infection time increases. **(C)** Immunoblot analysis of autophagy substrate in low-glucose (LG)-starved BMDMs treated with MHY1485 or bafilomycin A1 (Baf. A1). **(D)** Immunoblot analysis of autophagy substrate in Mtb-infected WT and SG^{neg} BMDMs or MHY1485-treated BMDMs. **(E)** Confocal microscopy images of tfLC3-transfected BMDMs. Cells were either infected with Mtb or starved in LG for 24 h. The arrowhead indicates the autolysosome. Scale bar indicates 10 µm. **(F)** Quantification of yellow puncta and red puncta from (E). The bar indicates the total number of yellow and red puncta in a cell. Each color represents a portion of yellow and red puncta. A total of 20 cells were analyzed. Data are presented as mean ± SD. n.s., non-significant, $^{**}p < 0.01$, compared with LG, using Kruskal–Wallis test followed by Dunn's post-hoc test. **(G)** Immunofluorescence analysis of LAMP1 (green) and LC3 (magenta) in RFP-Mtb-infected BMDMs treated with MHY1485 or rapamycin (Rapa.) for 24 h. The red dotted image indicates RFP-positive infected cells, while the white dotted image indicates RFP-negative uninfected cells. Nuclei: blue. Scale bar indicates 10 µm. Representative images are shown.

Figure S4. SG deficiency does not activate compensatory stress responses during Mtb infection

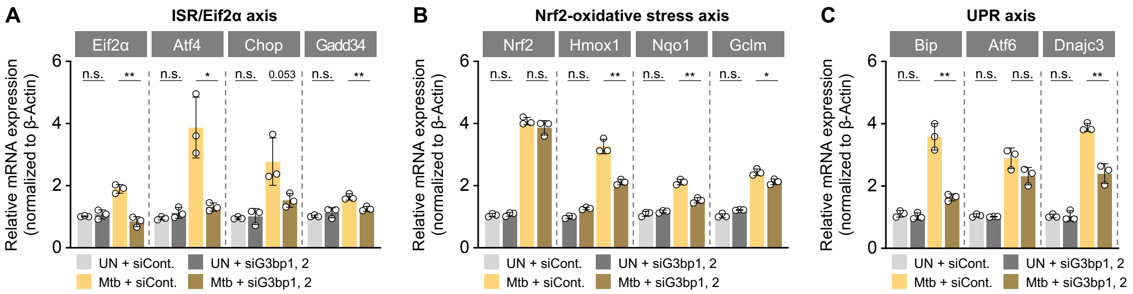

Figure S4. SG deficiency does not activate compensatory stress responses during Mtb infection
(A–C) Relative mRNA expression levels of genes involved in the ISR/Eif2α axis (A), Nrf2-mediated oxidative stress axis (B), and UPR axis (C) were measured in uninfected (UN) or Mtb-infected BMDMs transfected with siControl or siG3bp1, 2. Data are normalized to β-Actin and presendted as mean ± SD (n = 3). n.s., non-significant, *$p < 0.05$, **$p < 0.01$; *t*-test.

Figure S5. Double knockdown of G3bp1 and G3bp2 did not alter cap-dependent mRNA translation and immune responses of macrophages

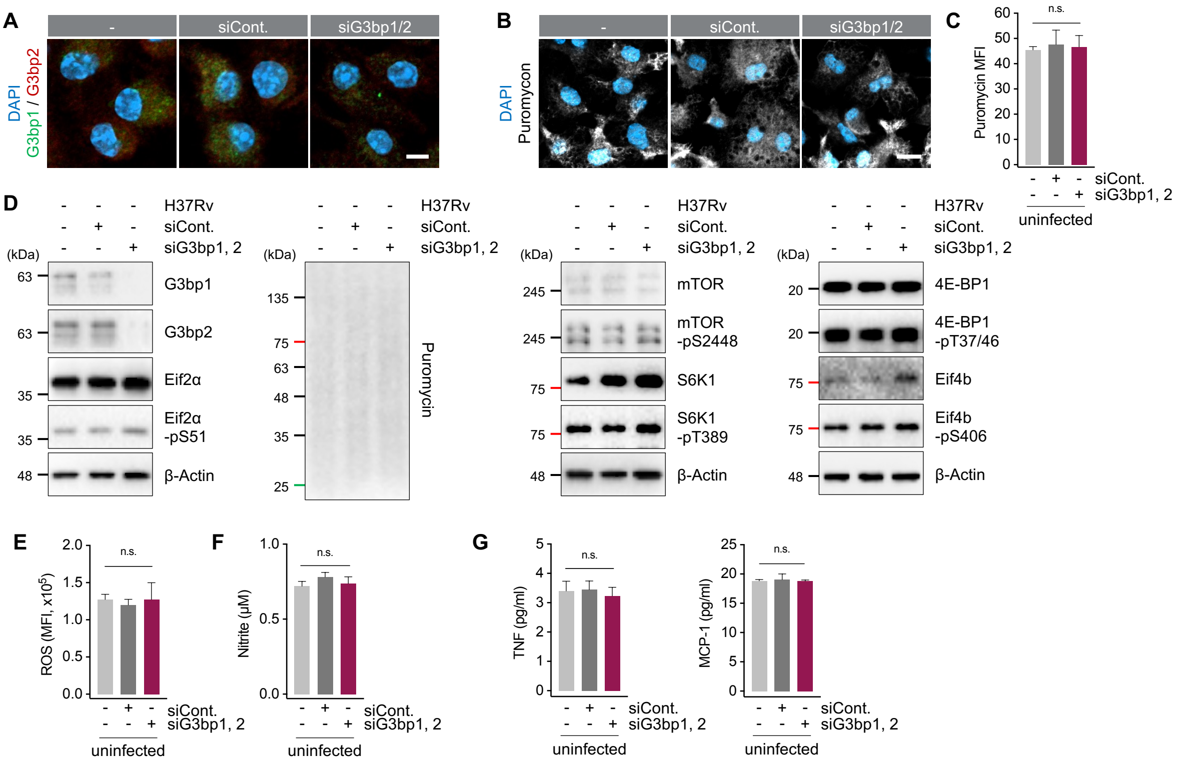

Figure S5. Double knockdown of G3bp1 and G3bp2 did not alter cap-dependent mRNA translation and immune responses of macrophages
(A) Immunofluorescence analysis of the uninfected G3bp1/2 dKD BMDMs. G3bp1 (green) and G3bp2 (red). Nuclei: blue. Scale bar indicates 5 μm. **(B)** Immunofluorescence analysis of ribopuromycylation in uninfected G3bp1/2 dKD BMDMs. Puromycin (white). Nuclei: blue. Scale bar indicates 10 μm. **(C)** Quantification of puromycin signal intensity from (B). **(D)** Immunoblot analysis of the uninfected G3bp1/2 dKD BMDMs. **(E)** Total ROS from uninfected BMDMs were measured by DHE staining. **(F)** Nitrite production was measured in uninfected BMDMs culture media. **(G)** Quantification of TNF and MCP-1 production in the uninfected BMDMs culture media.

Figure S6. SGs form independently of virulence and ROS, with minor antigen contribution

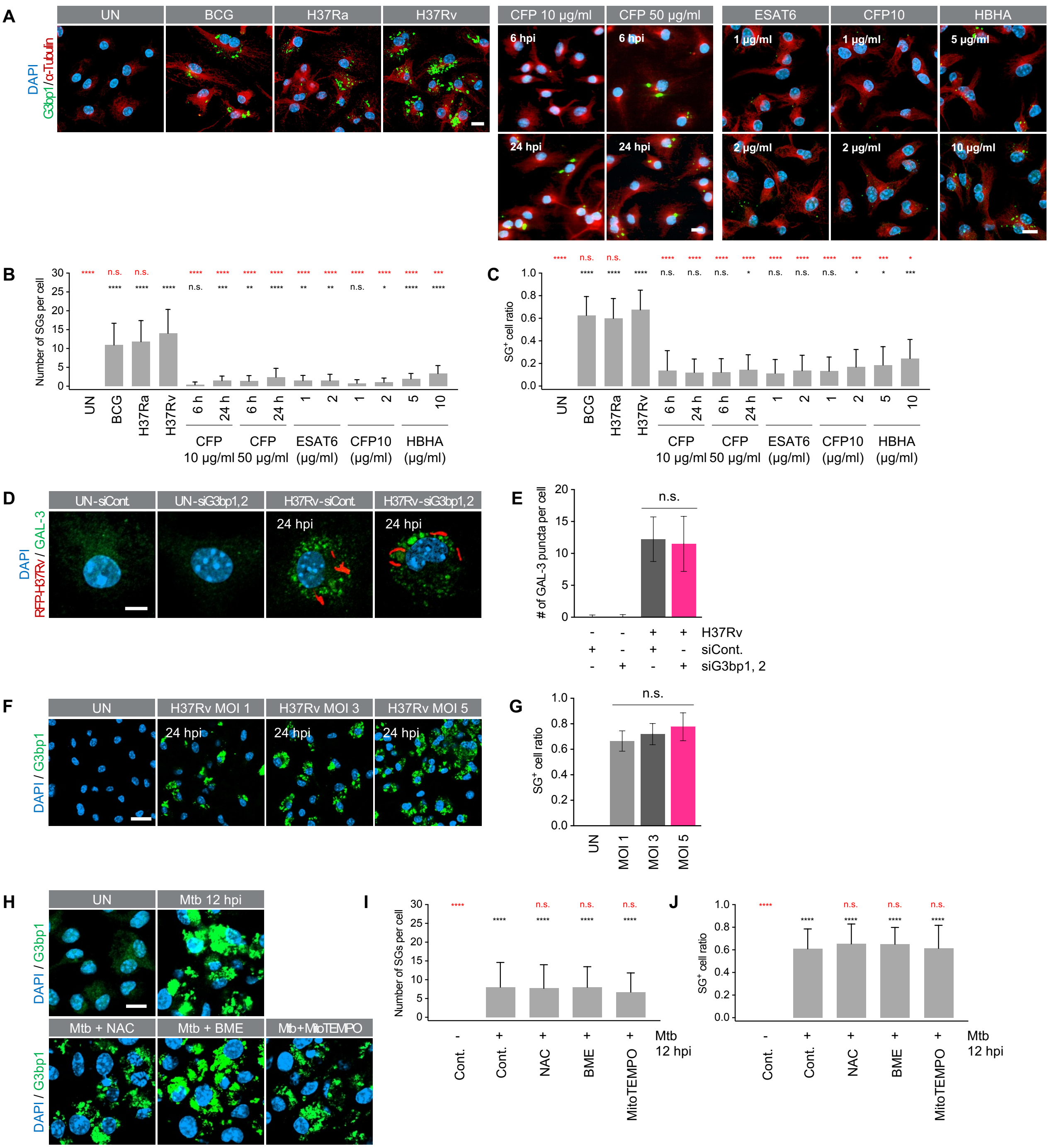

Figure S6. SGs form independently of virulence and ROS, with minor antigen contribution
(A) Immunofluorescence analysis of SGs in BMDMs infected by BCG, H37Ra, or H37Rv with a MOI of 1 for 24 h and treated with H37Rv culture filtrate proteins (CFPs), or viriulent antigens of H37Rv for 24 h. Nuclei: blue; G3bp1: green; α-Tubulin: red. Scale bar indicates 10 μm. **(B)** Quantification of number of SGs per cell from (A). n.s., non-significant, $*p < 0.05$, $**p < 0.01$, $***p < 0.001$, $****p < 0.0001$; compared with UN in black label; compared with H37Rv only in red label; One-way ANOVA followed by Tukey's multiple comparison post-hoc test. **(C)** Quantification of SG^+ cell ratio from (A). n.s., non-significant, $*p < 0.05$, $**p < 0.01$, $***p < 0.001$, $****p < 0.0001$; compared with UN in black label; compared with H37Rv only in red label; One-way ANOVA followed by Tukey's multiple comparison post-hoc test. **(D)** Immunofluorescence analysis of phagosomal damage using GAL-3 staining in siRNA-transfected BMDMs infected by RFP-H37Rv with a MOI of 1 for 24 h. Scale bar indicates 5 μm. **(E)** Quantification of number of GAL-3 puncta per cell from (D). n.s., non-significant; Mann-Whitney test. **(F)** Immuniflourescence analysis of SGs in BMDMs infected by H37Rv with a MOI of 1, 3, and 5 for 24 h. Nuclei: blue; G3bp1: green. Scale bar indicates 20 μm. **(G)** Quantification of SG^+ cell ratio from (F). n.s., non-significant. **(H)** Immunofluorescence analysis of SGs in H37Rv-infected BMDMs with ROS scavengers: NAC 0.5 mM, 2-mercaptoethanol (BME) 100 μM, and MitoTEMPO 100 μM. Nuclei: blue; G3bp1: green. Scale bar indicates 10 μm. **(I)** Quantification of number of SGs per cell from (H). n.s., non-significant, $****p < 0.0001$; compared with UN in black label; compared with H37Rv only in red label; One-way ANOVA followed by Tukey's multiple comparison post-hoc test. **(J)** Quantification of SG^+ cell ratio from (H). n.s., non-significant, $****p < 0.0001$; compared with UN in black label; compared with H37Rv only in red label; One-way ANOVA followed by Tukey's multiple comparison post-hoc test.

Figure S7. Inverse correlation between cellular ATP levels and SG dynamics

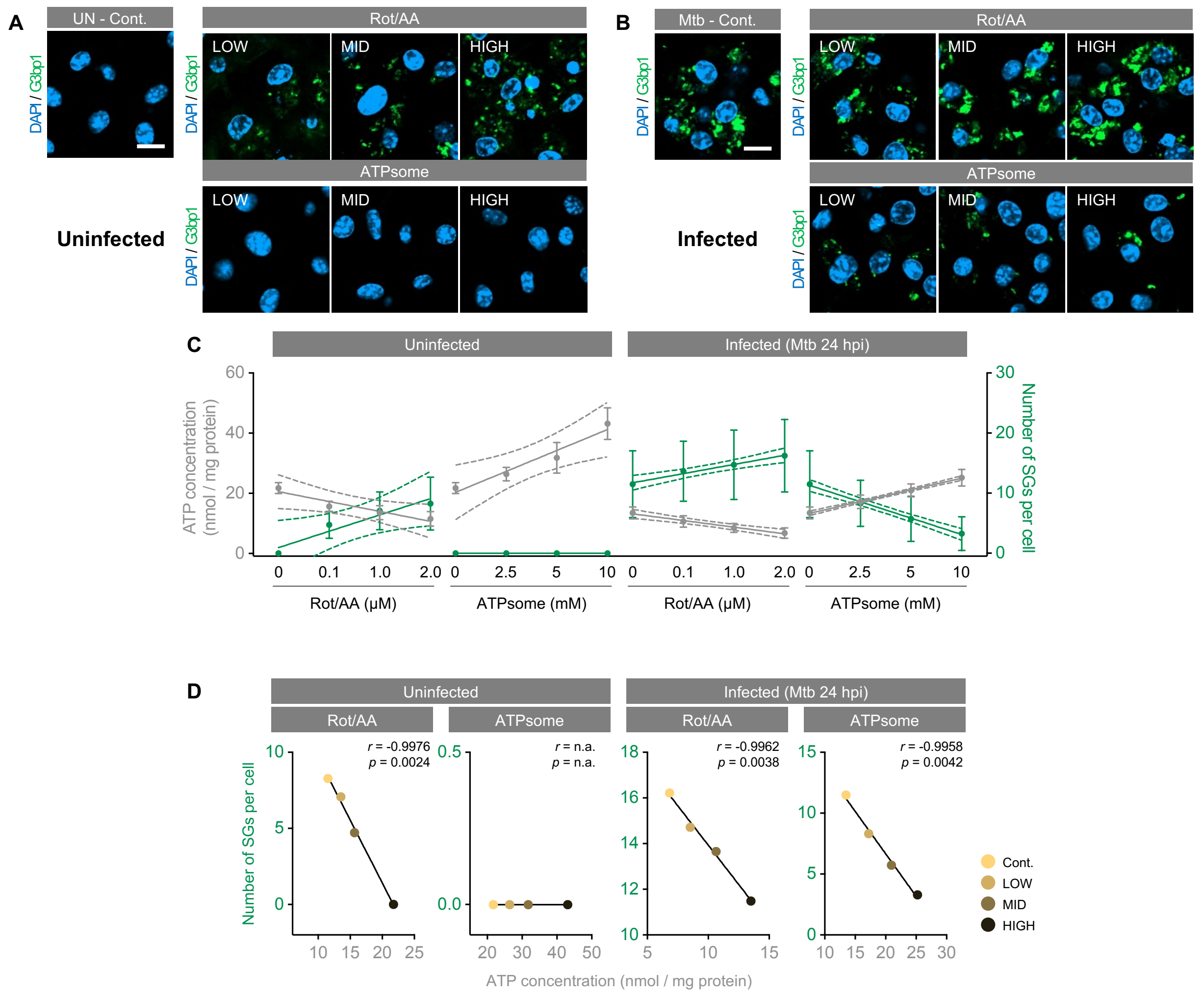

Figure S7. Inverse correlation between cellular ATP levels and SG dynamics

(A) Immunofluorescence analysis of uninfected BMDMs treated with Rot/AA (0.1 μM, LOW; 1.0 μM, MID; 2.0 μM, HIGH) or ATPsome (2.5 mM, LOW; 5.0 mM, MID; 10 mM, HIGH) for 24 h. Cells were stained for G3bp1 (green) and nuclei (blue). Scale bar 20 μm. **(B)** Immunofluorescence analysis of Mtb-infected BMDMs treated with Rot/AA (0.1 μM, LOW; 1.0 μM, MID; 2.0 μM, HIGH) or ATPsome (2.5 mM, LOW; 5.0 mM, MID; 10 mM, HIGH) for 24 h. Cells were stained for G3bp1 (green) and nuclei (blue). Scale bar 20 μm. **(C)** Quantification of intracellular ATP concentration (gray) and SG number per cell (green) under different conditions from (A and B). Data represent mean ± SD (n = 15 for ATP; n = 50 for SG). **(D)** Pearson correlation between ATP levels and SG number per cell for uninfected and Mtb-infected conditions from (A–C).

Figure S8. Lm infection induces ATP reduction and SG formation, thus inhibiting mTORC1 and innate immune activity

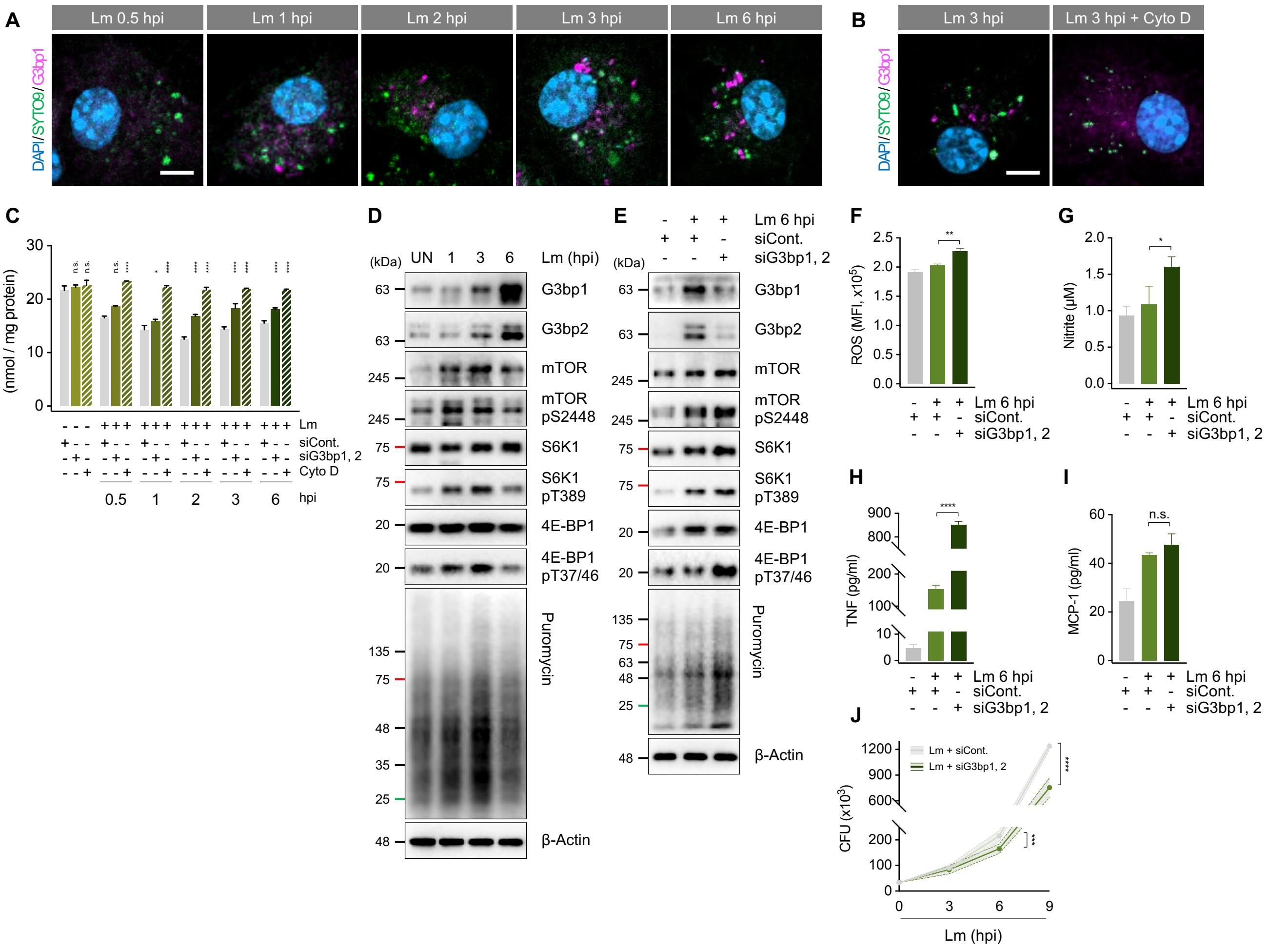

Figure S8. Lm infection induces ATP reduction and SG formation, thus inhibiting mTORC1 and innate immune activity
(A) Immunofluorescence analysis of the Lm-infected BMDMs. Cells were infected with Lm (MOI 1) and stained with SYTO9 (Lm) and G3bp1. Scale bar indicates 5 μm. **(B)** Immunofluorescence analysis of the Lm-infected BMDMs with or without cytochalasin D (Cyto D). Cells were infected with Lm (MOI 1) and stained with SYTO9 (Lm) and G3bp1. Scale bar indicates 5 μm. **(C)** Intracellular ATP concentration of Lm-infected WT and SGneg BMDMs and Cyto D-treated BMDMs. n.s., non-significant, *$p < 0.05$, ****$p < 0.0001$; compared with siCont; One-way ANOVA followed by Tukey's multiple comparison post-hoc test. **(D)** Immunoblot analysis of Lm-infected BMDMs undergoing the indicated time of infection. **(E)** Immunoblot analysis of Lm-infected WT and SGneg BMDMs. **(F)** Total amount of ROS was measured by dihydroethidium staining. WT and SGneg BMDMs were infected with Lm (MOI 1). **$p < 0.01$, unpaired *t*-test. **(G)** Nitrite production was measured in the Lm-infected WT and SGneg BMDM culture media. *$p < 0.05$, unpaired *t*-test. **(H and I)** Quantification of TNF (H) and MCP-1 (I) production in the Lm-infected WT and SGneg BMDM culture media. Data are presented as mean ± SD (n=3). n.s., non-significant, ****$p < 0.0001$; Mann–Whitney test. **(J)** CFU of Lm in WT and SGneg BMDMs. Data from three independent experiments are shown as mean ± SD. ***$p < 0.001$, ****$p < 0.0001$, compared within the same time point; Mann–Whitney test.
